# Renal arterial infusion of tempol prevents medullary hypoperfusion, hypoxia, and acute kidney injury in ovine Gram‐negative sepsis

**DOI:** 10.1111/apha.14025

**Published:** 2023-08-07

**Authors:** Ashenafi H. Betrie, Shuai Ma, Connie P. C. Ow, Rachel M. Peiris, Roger G. Evans, Scott Ayton, Darius J. R. Lane, Adam Southon, Simon R. Bailey, Rinaldo Bellomo, Clive N. May, Yugeesh R. Lankadeva

**Affiliations:** ^1^ Preclinical Critical Care Unit, Florey Institute of Neuroscience and Mental Health The University of Melbourne Melbourne Victoria Australia; ^2^ Translational Neurodegeneration Laboratory, Florey Institute of Neuroscience and Mental Health The University of Melbourne Melbourne Victoria Australia; ^3^ Division of Nephrology, Shanghai Ninth People's Hospital Shanghai Jiaotong University School of Medicine Shanghai China; ^4^ Biomedicine Discovery Institute and Department of Physiology Monash University Melbourne Victoria Australia; ^5^ Faculty of Veterinary and Agricultural Sciences The University of Melbourne Melbourne Victoria Australia; ^6^ Department of Critical Care, Melbourne Medical School The University of Melbourne Melbourne Victoria Australia; ^7^ Australian and New Zealand Intensive Care Research Centre Monash University Melbourne Victoria Australia; ^8^ Department of Intensive Care Austin Hospital Melbourne Victoria Australia; ^9^ Department of Intensive Care Royal Melbourne Hospital Melbourne Victoria Australia

**Keywords:** acute kidney injury, hypoxia, inflammation, nitric oxide synthase, renal microcirculation, sepsis

## Abstract

**Aim:**

Renal medullary hypoperfusion and hypoxia precede acute kidney injury (AKI) in ovine sepsis. Oxidative/nitrosative stress, inflammation, and impaired nitric oxide generation may contribute to such pathophysiology. We tested whether the antioxidant and anti‐inflammatory drug, tempol, may modify these responses.

**Methods:**

Following unilateral nephrectomy, we inserted renal arterial catheters and laser‐Doppler/oxygen‐sensing probes in the renal cortex and medulla. Noanesthetized sheep were administered intravenous (IV) *Escherichia coli* and, at sepsis onset, IV tempol (IVT; 30 mg kg^−1^ h^−1^), renal arterial tempol (RAT; 3 mg kg^−1^ h^−1^), or vehicle.

**Results:**

Septic sheep receiving vehicle developed renal medullary hypoperfusion (76 ± 16% decrease in perfusion), hypoxia (70 ± 13% decrease in oxygenation), and AKI (87 ± 8% decrease in creatinine clearance) with similar changes during IVT. However, RAT preserved medullary perfusion (1072 ± 307 to 1005 ± 271 units), oxygenation (46 ± 8 to 43 ± 6 mmHg), and creatinine clearance (61 ± 10 to 66 ± 20 mL min^−1^). Plasma, renal medullary, and cortical tissue malonaldehyde and medullary 3‐nitrotyrosine decreased significantly with sepsis but were unaffected by IVT or RAT. Consistent with decreased oxidative/nitrosative stress markers, cortical and medullary nuclear factor‐erythroid‐related factor‐2 increased significantly and were unaffected by IVT or RAT. However, RAT prevented sepsis‐induced overexpression of cortical tissue tumor necrosis factor alpha (TNF‐α; 51 ± 16% decrease; *p* = 0.003) and medullary Thr‐495 phosphorylation of endothelial nitric oxide synthase (eNOS; 63 ± 18% decrease; *p* = 0.015).

**Conclusions:**

In ovine Gram‐negative sepsis, renal arterial infusion of tempol prevented renal medullary hypoperfusion and hypoxia and AKI and decreased TNF‐α expression and uncoupling of eNOS. However, it did not affect markers of oxidative/nitrosative stress, which were significantly decreased by Gram‐negative sepsis.

## 1 INTRODUCTION

Acute kidney injury (AKI) develops in approximately 50% of patients with sepsis and is associated with increased morbidity and mortality.^1^, ^2^ Antibiotics, fluid resuscitation, and vasopressors are currently a cornerstone of septic AKI management, with renal replacement therapy recommended for severe AKI.^3^ However, there are no specific kidney‐protective therapies available. A better understanding of the pathophysiology of septic AKI may facilitate their development.

Sepsis‐induced hypoxia and inflammation are proposed to increase the bioavailability of reactive oxygen and nitrogen species in the kidney (i.e. renal oxidative/nitrosative stress).^4^, ^5^ Excessive generation of reactive oxygen and nitrogen species is attenuated by activation of antioxidant defense pathways such as nuclear factor‐erythroid‐related factor 2 (NRF2).^4^, ^6^ In sepsis, a dysregulation of this system may contribute to the renal medullary hypoperfusion and hypoxia, which precede the development of AKI^7^, ^8^, ^9^ and appears to contribute to its pathophysiology.^4^, ^10^ However, there has been limited progress in determining whether drugs that mitigate renal medullary tissue hypoxia can prevent or delay the progression of the renal dysfunction associated with septic AKI.^11^

Tempol (4‐hydroxy‐2,2,6,6‐tetramethylpiperadine‐1‐oxyl) is a water‐soluble synthetic heterocyclic nitroxide that can decrease oxidative stress and increase nitric oxide bioavailability.^12^, ^13^, ^14^ Tempol undergoes a rapid interconversion between three forms: the nitroxide, hydroxylamine (one‐electron reduced), and oxoammonium cation (one‐electron oxidized) in liver microsomes and various tissues.^12^, ^13^, ^14^ Tempol also has anti‐inflammatory properties, which are mediated by the inhibition of inflammatory cytokines production via nuclear factor‐kappa β and a decrease in tissue infiltration by innate immune cells.^12^, ^13^, ^14^ However, both the contribution of renal oxidative/nitrosative stress and inflammation to the development of septic AKI, and whether their deleterious effects on the renal medullary microcirculation can be mitigated by tempol, remains to be determined.

In ovine Gram‐negative sepsis, renal medullary tissue hypoxia occurs in the first hour after infection, which is 24 h prior to the development of AKI as characterized by oliguria, decreased creatinine clearance and elevated plasma creatinine.^7^, ^8^, ^9^ Accordingly, we tested the hypothesis that tempol, administered from the start of infusion of *Escherichia coli*, ameliorates renal medullary tissue hypoperfusion and hypoxia and prevents the development of AKI in a clinically relevant ovine model of Gram‐negative sepsis. To determine whether the mechanism of action of tempol is systemic or kidney‐specific, we compared the responses to an intravenous infusion of tempol and a 10‐fold lower dose of tempol infused directly into the renal artery in nonanesthetized healthy and septic sheep.

Moreover, to investigate the mechanisms mediating the effects of tempol in sepsis, we quantified plasma and renal tissue levels of a marker of lipid peroxidation (malondialdehyde [MDA]) and its counter‐regulatory NRF2‐dependent system, along with markers of inflammation (tumor necrosis factor alpha [TNF‐α]) and counter‐inflammation (interleukin‐10 [IL‐10]). We also assessed the expression of renal tissue concentrations of total nitrate and nitrite (NOx) and tissue markers of nitrosative stress (3‐nitrotyrosine [3‐NT]), endothelial and inducible isoforms of nitric oxide synthase (eNOS and iNOS), and the phosphorylation status of specific eNOS amino acids, which determines whether eNOS produces nitric oxide (i.e. coupled, phosphorylated Ser‐1177) or decreases its bioavailability (i.e. uncoupled, phosphorylated Thr‐495).

## 2 RESULTS

### 2.1 Effects of tempol in healthy sheep

In healthy sheep, administration of intravenous tempol (IVT) or renal arterial tempol (RAT) over 4 h had no significant effects on systemic and renal hemodynamics, renal cortical and medullary tissue perfusion and oxygenation or kidney function (Figure S1).

### 2.2 Pharmacokinetics of IV and renal arterial infusion of tempol in healthy sheep

Continuous IV infusion of tempol (30 mg kg^−1^ h^−1^) for 4 h resulted in a higher concentration of total tempol (1.09 ± 0.23 vs. 0.07 ± 0.04 mmol L^−1^) and tempol nitroxide radical (0.81 ± 0.27 vs. 0.12 ± 0.12 mmol L^−1^) in arterial blood compared with the renal arterial route of administration (3 mg kg^−1^ h^−1^) by the end of the 4th hour (Figure S2). After stopping the continuous infusion, we took plasma samples until 6 h to determine pharmacokinetic parameters. A one‐compartmental pharmacokinetic model showed that the tempol nitroxide radical was eliminated at a faster rate (0.73 vs. 0.40 h^−1^), had a higher clearance rate (0.03 vs. 0.02 mg kg^−1^ μmol L^−1^ h^−1^) and had a shorter half‐life (0.95 vs. 1.73 h) compared with total tempol (Table S1). There was a higher concentration of the nitroxide tempol radical in renal venous blood from the renal arterial route compared with the IV route of administration (Figure S2).

### 2.3 Systemic hemodynamics

**FIGURE 1:**
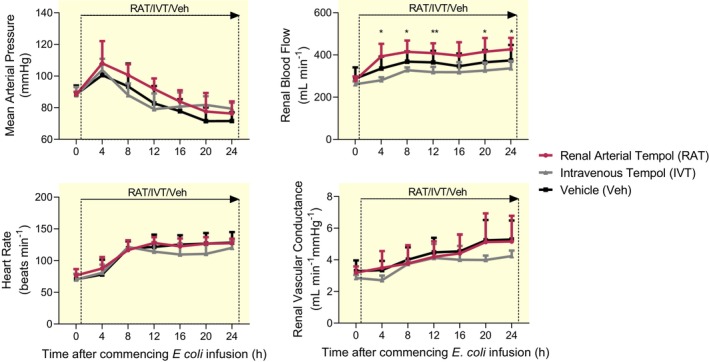
Systemic and renal hemodynamics during renal arterial infusion of tempol or intravenous infusion of tempol or vehicle during 24 h of ovine Gram‐negative sepsis. Mean arterial pressure, renal blood flow, heart rate and renal vascular conductance during a 24 h continuous infusion of live *Escherichia coli*. Simultaneously, each conscious sheep also received either a renal arterial infusion of tempol (RAT; *n* = 7) or vehicle (Veh; *n* = 7) or an intravenous infusion of tempol (IVT; *n* = 6). Data are mean ± SD. Time 0 is the mean of the 24th h of the baseline period, and times 4–24 are means of 1‐h periods. Data were analyzed using a two‐way repeated measures ANOVA with factors ‘group’ (*P*_Group_), ‘time’ (*P*_Time_), and their interaction (*P*_Group × Time_), then a Tukey's post‐test was performed to adjust *p* values for making between‐group multiple comparisons between vehicle, renal arterial tempol and intravenous tempol groups at each of the sepsis‐time points. *p ≤ 0.05 for comparison between sheep treated with a renal arterial infusion of tempol and those receiving an intravenous infusion of tempol (Tukey's post‐test).

In vehicle‐treated sheep, *E. coli* infusion was accompanied by progressive hypotension and tachycardia over 24 h (Figure 1). These responses were not significantly affected by IVT or RAT treatment.

### 2.4 Renal hemodynamics and oxygen handling

**FIGURE 2:**
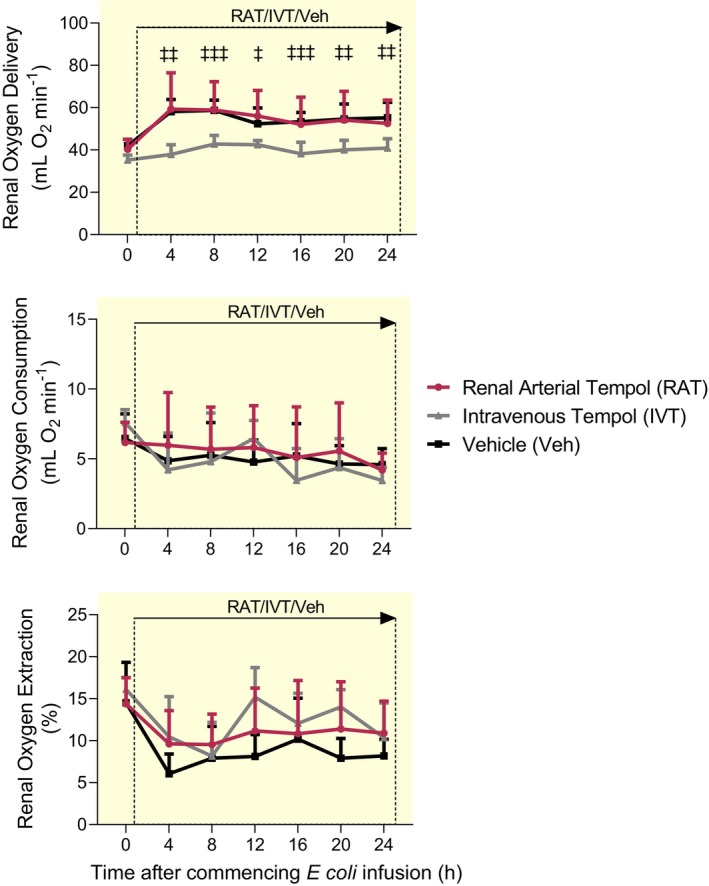
Global renal oxygen handling during renal arterial infusion of tempol or intravenous infusion of tempol or vehicle during 24 h of ovine Gram‐negative sepsis. Renal oxygen delivery, renal oxygen consumption and renal oxygen extraction during a 24 h continuous infusion of live *Escherichia coli*. Simultaneously, each conscious sheep also received either a renal arterial infusion of tempol (RAT; *n* = 6) or vehicle (Veh; *n* = 6) or an intravenous infusion of tempol (IVT; *n* = 6). Data are means ± SD. Time 0 is the mean of the 24th hour of the baseline period, and times 4–24 are means of 1‐h periods. Data were analyzed using a two‐way repeated measures ANOVA with factors ‘group’ (*P*_Group_), ‘time’ (*P*_Time_) and their interaction (*P*_Group × Time_), then a Tukey's post‐test was performed to adjust *p* values for making between‐group multiple comparisons between vehicle, renal arterial tempol and intravenous tempol groups at each of the sepsis‐time points. ^‡^ *p* ≤ 0.05, ^‡‡^ *p* ≤ 0.01, ^‡‡‡^ *p* ≤ 0.001 for comparison between sheep treated with an intravenous infusion of tempol and those receiving a renal arterial infusion of vehicle (Tukey's post‐test).

*Escherichia coli* infusion was accompanied by significantly increased renal blood flow (RBF) and renal vascular conductance (RVC) across all groups (Figure 1). There was, however, a significantly greater increase in RBF with RAT than with IVT. Global renal hyperemia was accompanied by increased renal oxygen delivery (RDO_2_; 41 ± 14 to 49 ± 17 mL O_2_ min^−1^) in vehicle‐treated sheep, an effect attenuated by IVT (35 ± 2 to 40 ± 2 mL O_2_ min^−1^; Figure 2). However, neither renal oxygen consumption (RVO_2_) nor renal oxygen extraction was significantly affected by IVT or RAT over 24 h (Figure 2).

### 2.5 Intra‐renal tissue perfusion and oxygenation

**FIGURE 3:**
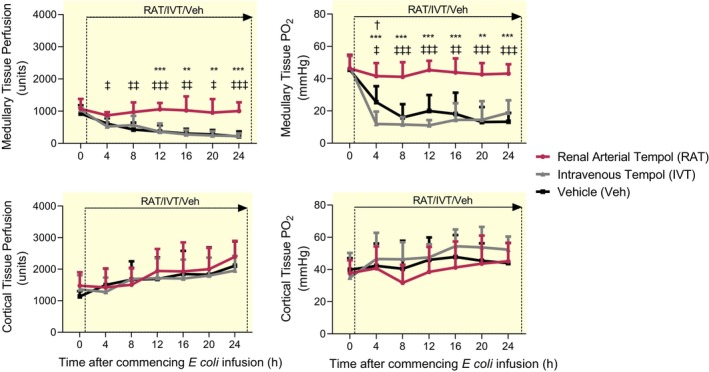
Renal tissue perfusion and oxygen tension during renal arterial infusion of tempol or intravenous infusion of tempol or vehicle for 24 h ovine Gram‐negative sepsis. Medullary tissue perfusion, medullary tissue oxygen tension (PO_2_), cortical tissue perfusion and cortical tissue PO_2_ during a 24 h continuous infusion of live *Escherichia coli*. Simultaneously, each conscious sheep also received either a renal arterial infusion of tempol (RAT; *n* = 7) or vehicle (Veh; *n* = 7) or an intravenous infusion of tempol (IVT; *n* = 6). Data are expressed as mean ± SD. Time 0 is the mean of the 24th hour of the baseline period, and times 4–24 are means of 1‐h periods. Data were analyzed using a two‐way repeated measures ANOVA with factors ‘group’; (*P*_Group_), ‘time’ (*P*_Time_), and their interaction (*P*_Group × Time_), then a Tukey's post‐test was performed to adjust *p* values for making between‐group multiple comparisons between vehicle, renal arterial tempol and intravenous tempol groups at each of the sepsis‐time points. ^‡^ *p* ≤ 0.05, ^‡‡^ *p* ≤ 0.01, ^‡‡‡^ *p* ≤ 0.001 for comparison between sheep treated with a renal arterial infusion of tempol and those receiving a renal arterial infusion of vehicle (Tukey's post‐test). **p ≤ 0.01, ***p ≤ 0.001 for comparison between sheep treated with a renal arterial infusion of tempol and those receiving an intravenous infusion of tempol (Tukey's post‐test). ^†^ *p* ≤ 0.05 for comparison between sheep treated with intravenous infusion of tempol and those receiving a renal arterial infusion of vehicle (Tukey's post‐test).

In vehicle‐treated sheep, renal medullary tissue perfusion (965 ± 292 to 613 ± 170 units) and oxygen tension (PO_2_; 46 ± 9 to 25 ± 10 mmHg) decreased after 4 h of *E. coli* infusion, with a further decrease to 216 ± 154 units and 13 ± 6 mmHg, respectively, after 24 h (Figure 3). IVT did not significantly modify the changes in medullary perfusion (1035 ± 90 to 224 ± 88 units) and PO_2_ (49 ± 6 to 19 ± 8 mmHg) after 24 h of sepsis. In contrast, RAT significantly attenuated the sepsis‐induced reductions in renal medullary perfusion (1072 ± 308 to 1005 ± 271 units) and PO_2_ (47 ± 8.3 to 43 ± 6 mmHg) during the 24 h of sepsis (Figure 3). These changes in renal medullary tissue oxygenation with vehicle, RAT and IVT, were closely mirrored by changes in bladder urinary oxygenation (*r* ^2^ = 0.456; Figure S3).

In vehicle‐treated sheep, renal hyperemia was accompanied by a progressive increase in renal cortical perfusion (1134 ± 166 to 2110 ± 769 units) without a significant change in cortical tissue PO_2_ (40 ± 7 to 44 ± 13 mmHg; Figure 3). RAT or IVT did not significantly modify these changes.

### 2.6 Renal function

**FIGURE 4:**
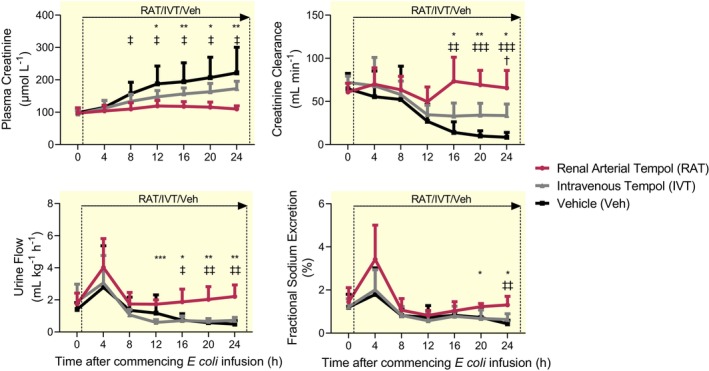
Renal function during renal arterial infusion of tempol or intravenous infusion of tempol or vehicle for 24 h ovine Gram‐negative sepsis. Plasma creatinine, creatinine clearance, urine flow and fractional sodium excretion during a 24 h infusion of live *Escherichia coli*. Simultaneously, each conscious sheep also received either a renal arterial infusion of tempol (RAT; *n* = 7) or vehicle (Veh; *n* = 7) or an intravenous infusion of tempol (IVT; *n* = 6) from 0 to 24 h in conscious sheep. Data are expressed as mean ± SD. Time 0 is the mean of the 24th hour of the baseline period, and times 4–24 are means of 1‐h periods. Data were analyzed using a two‐way repeated measures ANOVA with factors ‘group’ (*P*_Group_), ‘time’ (*P*_Time_), and their interaction (*P*_Group × Time_), then a Tukey's post‐test was performed to adjust *p* values for making between‐group multiple comparisons between vehicle, renal arterial tempol and intravenous tempol groups at each of the sepsis‐time points. *p ≤ 0.05, **p ≤ 0.01, ***p ≤ 0.001 for comparison between sheep treated with a renal arterial infusion of tempol and those receiving an intravenous infusion of tempol (Tukey's post‐test). ^‡^ *p* ≤ 0.05, ^‡‡^ *p* ≤ 0.01, ^‡‡‡^ *p* ≤ 0.001 for comparison between sheep treated with a renal arterial infusion of tempol and those receiving a renal arterial infusion of vehicle (Tukey's post‐test). ^†^ *p* ≤ 0.05 for comparison between sheep treated with intravenous infusion of tempol and those receiving a renal arterial infusion of vehicle (Tukey's post‐test).

In vehicle‐treated sheep, AKI developed after 24 h of sepsis with increased plasma creatinine (98 ± 9 to 222 ± 79 μmol L^−1^) and decreased urine flow (1.4 ± 0.7 to 0.5 ± 0.2 mL kg^−1^ h^−1^), creatinine clearance (65 ± 18 to 8 ± 6 mL min^−1^), and fractional excretion of sodium (1.2 ± 0.5 to 0.4 ± 0.2%; Figure 4). IVT did not affect the development of septic AKI. In contrast, RAT preserved plasma creatinine concentration (97 ± 16 to 105 ± 10 μmol L^−1^), urine flow (1.8 ± 0.7 to 2.2 ± 0.7 mL kg^−1^ h^−1^), creatinine clearance (61 ± 10 to 66 ± 20 mL min^−1^), and fractional sodium excretion (1.4 ± 0.6 to 1.3 ± 0.4%; Figure 4).

### 2.7 Plasma MDA and renal tissue expression of MDA, 3‐NT, and NRF2

**FIGURE 5:**
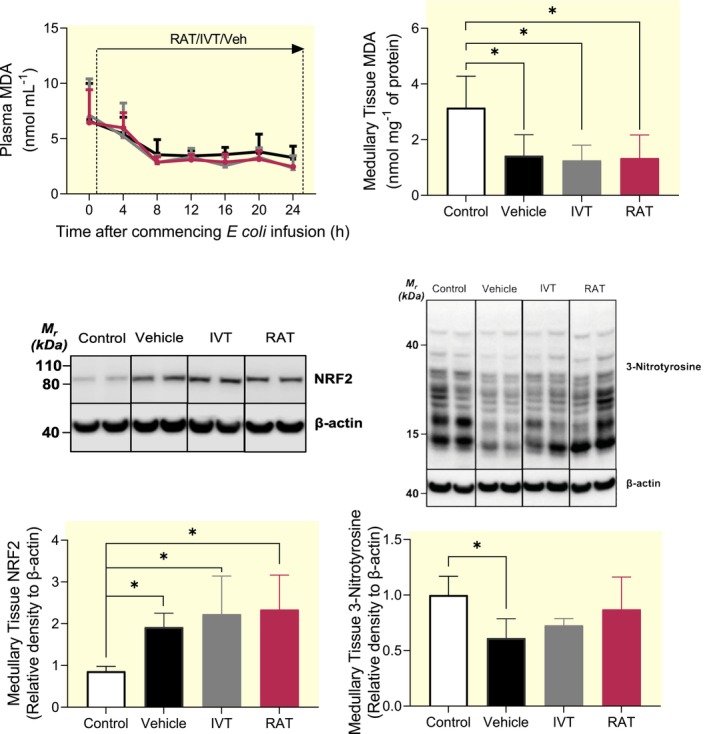
Plasma markers of oxidative stress and renal medullary tissue markers of tissue oxidative stress, nitrosative stress and antioxidant defense mechanisms. Plasma malonaldehyde (MDA) in septic sheep treated either with a renal arterial infusion of tempol (RAT; *n* = 7) or vehicle (*n* = 7) or an intravenous infusion of tempol (IVT; *n* = 6) during the 24 h of sepsis. Data were analyzed using a two‐way repeated measures ANOVA with factors ‘group’ (*P*_Group_), ‘time’ (*P*_Time_) and their interaction (*P*_Group × Time_), then a Tukey's post‐test was performed to adjust *p* values for making between‐group multiple comparisons between vehicle, renal arterial tempol and intravenous tempol groups at each of the sepsis‐time points. Medullary MDA, medullary nuclear factor‐erythroid related factor 2, and medullary 3‐nitrotyrosine in renal tissue derived from those septic sheep, at the end of the 24 h period of sepsis, and from a group of naïve sheep (control; *n* = 5). Values are mean ± SD of protein expression relative to the expression of β‐actin. Data were analyzed using a one‐way ANOVA and *p ≤ 0.05 was derived from a Tukey's post‐test for comparisons between the different treatment groups.

Plasma MDA concentration progressively fell in vehicle‐treated septic sheep (6.5 ± 3.0 to 2.5 ± 0.2 nmol mL^−1^), a change unaffected by IVT or RAT (Figure 5).

Healthy (control) sheep had higher medullary tissue MDA expression (3.1 ± 1.1 nmol mg^−1^ of protein) compared with vehicle‐treated septic sheep (1.4 ± 0.8 nmol mg ^−1^ of protein; *p* = 0.024) and sheep receiving RAT (1.3 ± 0.8 nmol mg^−1^ of protein; *p* = 0.016) or IVT (1.3 ± 0.5 nmol mg^−1^ of protein; *p* = 0.020; Figure 5). A similar pattern was seen for cortical tissue MDA concentration (Figure S4).

Compared with healthy sheep, the medullary tissue levels of the master regulator of antioxidant responses, NRF2, were significantly greater in septic sheep treated with vehicle (+132 ± 34%; *p* = 0.046), RAT (+212 ± 112%; *p* = 0.004) and IVT (+154 ± 84%; *p* = 0.014; Figure 5). A similar pattern was seen for cortical tissue NRF2 (Figure S4).

Medullary tissue 3‐NT expression was less (−39 ± 18%; *p* = 0.018) in vehicle‐treated septic sheep than in healthy sheep (Figure 5). The mean decrease in medullary 3‐NT in IVT and RAT sheep was not significantly different than those in vehicle‐treated sheep. Cortical tissue 3‐NT concentration was similar across the groups (Figure S4).

### 2.8 Plasma and renal tissue expression of TNF‐α and IL‐10

**FIGURE 6:**
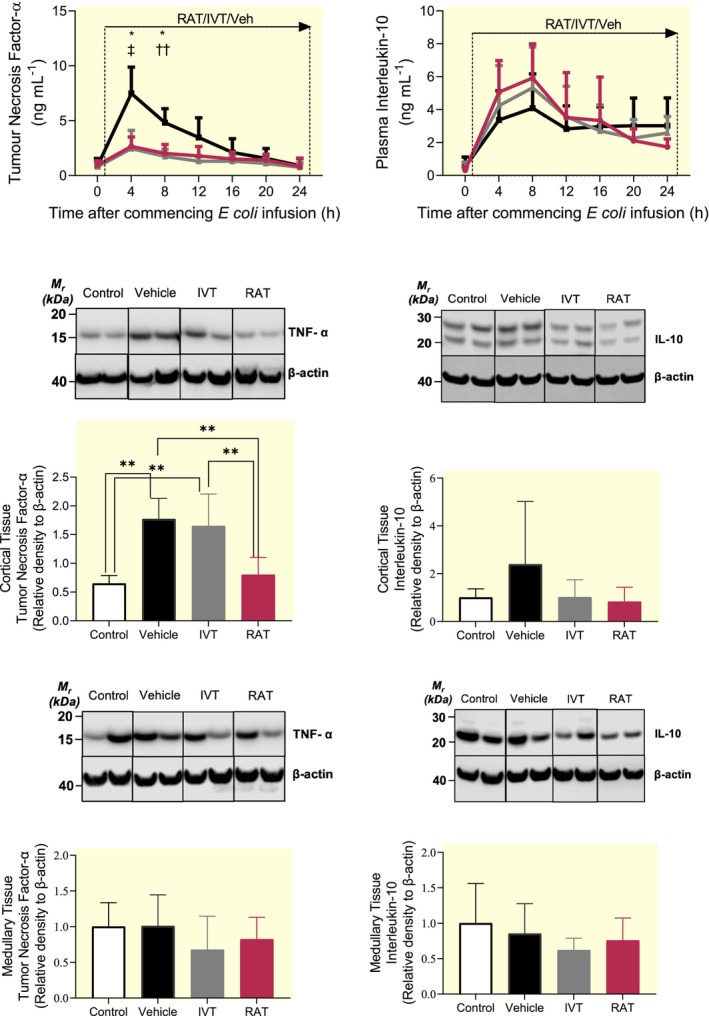
Plasma cytokines and the protein expression of renal cortical and medullary tissue cytokines. Plasma tumor necrosis factor alpha (TNF‐α) and plasma interleukin‐10 (IL‐10) in septic sheep treated either with a renal arterial infusion of tempol (RAT; *n* = 7) or vehicle (*n* = 7) or an intravenous infusion of tempol (IVT; *n* = 5) during the 24 h period of sepsis. Cortical TNF‐α, cortical IL‐10, medullary TNF‐α and medullary IL‐10 in renal tissue derived from those septic sheep, at the end of the 24 h period of sepsis, and also from a group of naive sheep (control; *n* = 5). Values presented as mean ± SD of protein expression relative to the expression of β‐actin. Data were analyzed using a one‐way ANOVA and *p ≤ 0.05 and **p ≤ 0.01 are derived from a Tukey's post‐test for comparisons between the different treatment groups.

The plasma concentration of TNF‐α increased from 0.9 ± 0.4 to 7.5 ± 2.4 ng mL^−1^ after 4 h of sepsis in the vehicle‐treated group, prior to returning toward premorbid levels by 24 h of sepsis (0.9 ± 0.2 ng mL^−1^; Figure 6). IVT and RAT significantly attenuated the magnitude of this TNF‐α response. The plasma concentration of IL‐10 increased to peak levels by 8 h of sepsis (0.5 ± 0.6 to 4.1 ± 2.1 ng mL^−1^) and remained elevated at 24 h in vehicle‐treated sheep (3.1 ± 1.7 ng mL^−1^) and was not affected by RAT and IVT (Figure 6).

Renal cortical tissue TNF‐α expression was greater in vehicle (107 ± 53%; *p* = 0.008) and IVT (110 ± 71%; *p* = 0.011) treated septic sheep than in healthy controls (Figure 6). In contrast, RAT suppressed the elevation in cortical TNF‐α concentration to control levels (Figure 6). In all three groups, renal cortical expression of IL‐10, and renal medullary tissue expression of TNF‐α and IL‐10, were similar to the levels in controls (Figure 6).

### 2.9 Renal tissue expression of NOS isoforms

**FIGURE 7:**
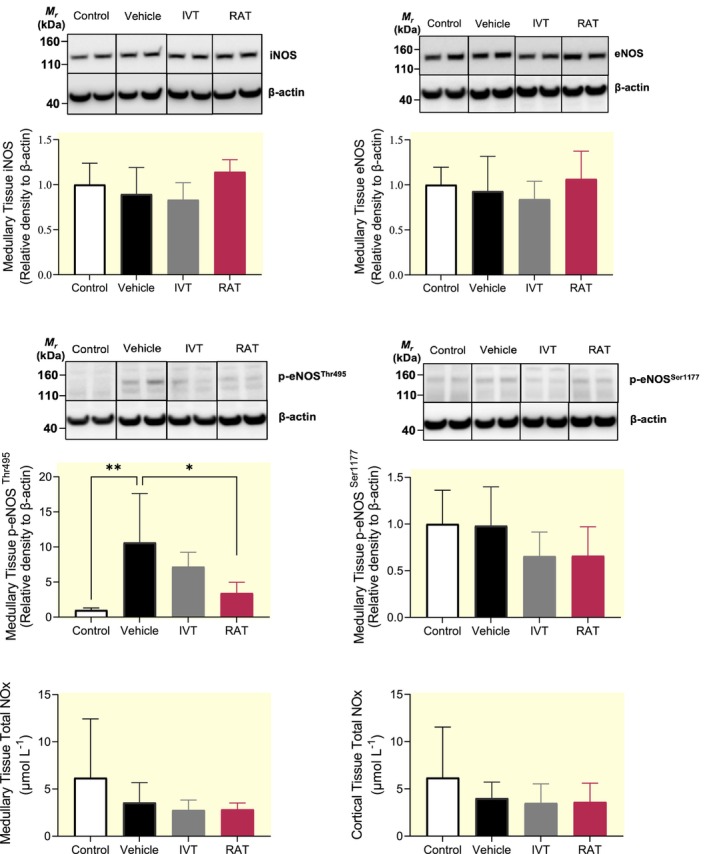
Renal medullary tissue protein expression of nitric oxide synthase isoforms and renal tissue concentrations of total nitric oxide metabolites. Medullary inducible nitric oxide synthase (iNOS), medullary endothelial nitric oxide synthase (eNOS), medullary eNOS phosphorylated at threonine at position 495 (p‐eNOS^Thr495^), and medullary eNOS phosphorylated at serine at position 1177 (p‐eNOS^Ser‐1177^) in renal tissue derived from naïve sheep (control; *n* = 5) and from septic sheep treated either with a renal arterial infusion of tempol (RAT; *n* = 7) or vehicle (*n* = 7) or an intravenous infusion of tempol (IVT; *n* = 5) during the 24 h period of sepsis. Data are expressed as mean ± SD of protein expression relative to the expression of β‐actin. Data were analyzed using a one‐way ANOVA and *p ≤ 0.05, **p ≤ 0.01, and ***p ≤ 0.001 are derived from a Tukey's post‐test for comparisons between the different treatment groups.

Renal medullary tissue levels of total iNOS, total eNOS, and Ser‐1177 phosphorylated eNOS (i.e., “activating/coupling” site) were not significantly affected by sepsis or RAT or IVT (Figure 7). However, renal medullary expression of Thr‐495 phosphorylated eNOS (i.e., “inhibitory/uncoupling” site) was significantly upregulated in vehicle‐treated septic sheep compared with controls (+964 ± 450%; *p* = 0.002; Figure 7). This increase was significantly decreased by RAT (+282 ± 190%; *p* = 0.015), with a trend toward an attenuation by IVT (+550 ± 179%; *p* = 0.2; Figure 7). NOx concentrations in renal cortical and medullary tissue were not significantly different between treatment groups (Figure 7).

Cortical tissue levels of iNOS, eNOS, and Ser‐1177 phosphorylated eNOS were not significantly different in controls compared with IVT‐ or RAT‐treated septic sheep (Figure S5). Thr‐495 phosphorylated eNOS could not be detected within the renal cortex (data not presented).

## 3 DISCUSSION

This is the first study, conducted in a large mammal, to demonstrate that tempol administered directly into the renal artery prevented the onset of renal medullary hypoperfusion and hypoxia and the development of AKI over a clinically appropriate time period of 24 h. However, these benefits in septic sheep achieved with a renal arterial infusion of tempol were not observed when a 10‐fold higher dosage was administered intravenously. The protective effects of tempol, when administered directly into the kidney, occurred in the absence of an overexpression or activation of markers of systemic or renal oxidative/nitrosative stress, which decreased during sepsis. Instead, infusion of tempol directly into the renal artery prevented sepsis‐induced upregulation of TNF‐α in cortical tissue and uncoupling of eNOS in medullary tissue. Tempol had no effects on renal perfusion and function in healthy sheep independent of the route of administration, which suggests that the effects of its renal arterial infusion result from an antagonistic action on the pathophysiology of septic AKI.

We found that selective decreases in perfusion and oxygenation within the renal medulla preceded the development of AKI, which is similar to our previous findings in this ovine model of Gram‐negative hyperdynamic sepsis.^7^, ^8^, ^9^ Such renal medullary microcirculatory dysfunction in ovine sepsis occurs despite an increase in total RBF and RDO_2_ and preserved renal cortical tissue perfusion and oxygenation, suggesting that it results from redistribution of intra‐RBF (shunting). In healthy nonanesthetized sheep, there is no significant difference in tissue perfusion and oxygenation between the renal cortex and medulla.^8^, ^9^ However, the renal medulla appears to be particularly susceptible to developing hypoxia under pathophysiological conditions including sepsis^7^, ^8^, ^9^ and cardiopulmonary bypass.^15^, ^16^, ^17^ Such deficit in the autoregulatory capacity of the medullary circulation relative to the cortical circulation has also been demonstrated in response to reductions in RBF by 20% and 50% induced by renal artery occlusion in nonanesthetized sheep.^18^ However, whether renal medullary hypoxia is correlative or causative of the functional deficits of AKI has not been previously investigated.^11^ The critical test of this hypothesis is to examine whether amelioration of the early onset of renal medullary hypoxia can prevent the development of septic AKI.

In the current study, renal arterial infusion of tempol prevented the onset of renal medullary tissue hypoperfusion and hypoxia and protected against the renal functional deficits of AKI over 24 h of Gram‐negative sepsis. Renal tissue hypoxia can generate superoxide radicals causing tonic constriction of the vasa recta leading to progressive medullary hypoperfusion.^4^, ^19^ The unique characteristic of tempol lies in its complex redox activities, where the nitroxide radical acts as a mimetic for superoxide dismutase by scavenging superoxide, curbing the formation of hydroxyl radicals, and reducing the intracellular concentration of labile Fe(II).^13^, ^20^ In agreement, direct administration of tempol into the renal medullary vasa recta induced nitric oxide‐mediated vasodilation.^21^ Our proof‐of‐concept pharmacokinetic study in healthy sheep demonstrates that, at the doses used, the renal arterial route of administration delivers a numerically higher concentration of the nitroxide tempol radical to the kidneys compared with the in route. It is conceivable that a higher concentration of the nitroxide radical of tempol is required in sepsis to dismutate superoxide radicals and augment nitric oxide bioavailability in the renal medulla under hypoxic conditions. This may partly explain why we did not observe similar reno‐protective effects with IV tempol (IVT) treatment in septic sheep. A limitation in our pharmacokinetic study was the inability to perform statistical analysis due to the loss of patency of the renal venous catheters surgically implanted in two out of the five healthy sheep. We also found that neither renal arterial nor IV infusion of tempol had any significant effects on renal perfusion or oxygenation or kidney function in healthy sheep. Collectively, our findings suggest that the benefits achieved by a renal arterial infusion of tempol on the renal medullary microcirculation and kidney function result from its antagonistic actions on the pathophysiology of septic AKI.

An important observation in our study was that only direct renal arterial, but not IV, infusion of tempol prevented the overexpression of renal cortical tissue TNF‐α protein seen in ovine septic AKI. In sepsis, TNF‐α production within the kidneys is mediated by the infiltration of innate immune cells including neutrophils.^22^ In accord, TNF‐α expression is increased in the glomeruli and proximal convoluted tubules, mainly located within the renal cortex, in rodent models of sepsis.^23^ In ovine sepsis, we previously reported a higher degree of neutrophil infiltration in the renal cortex,^24^ which may be a consequence of the redistribution of blood perfusion away from the medulla. The enhanced neutrophil sequestration may explain the selective up‐regulation of TNF‐α levels in renal cortical compared with medullary tissue in septic sheep. The higher delivery of the tempol nitroxide radical via the renal arterial route of administration may have led to the reduction in renal cortical TNF‐α by an attenuation of nuclear factor‐kappa B and decreased tissue infiltration of innate immune cells.^25^, ^26^ The inability to assess the direct interaction between TNF‐α concentrations in tissue and innate immune cell function is a limitation of the current experimental design, which warrants more detailed mechanistic investigation in future studies.

We found selective upregulation of renal medullary tissue Thr‐495 phosphorylated eNOS protein in ovine septic AKI, a well‐established contributor to the uncoupling of eNOS, which decreases nitric oxide synthesis and signaling.^4^, ^27^, ^28^ We further found that direct renal arterial infusion of tempol prevented the uncoupling of eNOS by attenuating the expression of renal medullary tissue Thr‐495 phosphorylated eNOS protein, a form of eNOS that diminishes NO bioavailability and increases superoxide producing activities.^29^ Similarly, tempol has been reported to preserve renal tissue nitric oxide bioavailability in rodent models of ischemia reperfusion injury.^30^, ^31^ Thus, improved bioavailability/signaling of nitric oxide may also contribute to the preserved renal medullary perfusion and PO_2_ observed when tempol was administered directly into the renal circulation. Future studies are required to determine whether a renal arterial infusion of tempol in late stages of established ovine septic AKI can reverse the renal functional deficits.

In our study, the development of AKI was not associated with increased markers of oxidative stress, rather these markers were decreased. Similarly, we previously reported a decrease in another lipid peroxidation marker, urinary F_2_‐isoprostane, in ovine Gram‐negative septic AKI.^32^ In human sepsis, increased serum MDA levels have been correlated with increased morbidity and mortality.^33^, ^34^, ^35^ However, clinical studies lack a reference group to establish whether MDA levels rise during sepsis, since the onset of infection is unknown, and pre‐morbid baseline levels cannot be determined. Indeed, the purported rise in MDA levels in human sepsis from ~1 to 3 nmol/mL^33^, ^34^, ^35^ is ~50% lower than the pre‐morbid levels seen in sheep. Moreover, the peak serum MDA concentration reported in human sepsis (~2.5–3.0 nmol mL^−1^) is similar in magnitude to the temporally diminishing plasma MDA levels in septic sheep at 24 h of infection. Our findings challenge the current narrative in sepsis,^4^, ^5^ but they are consistent with renal hypoxia decreasing the availability of oxygen to participate in redox chemistry. Notably, in our study, the decrease in renal tissue MDA was accompanied by an increase in renal tissue NRF2 expression, which could help explain the decrease in oxidative stress markers. The importance of the renal NRF2 pathway in protecting the kidneys from sepsis‐induced oxidative stress and renal injury is a consistent finding in rats subjected to endotoxemia or cecal ligation and punction.^36^, ^37^

We found upregulation in the renal cortical and medullary tissue expression of NRF2 and a lower protein expression of markers of nitrosative stress (3‐NT and iNOS) in septic sheep. The generation of nitrosative radicals requires the presence of high levels of both superoxide and nitric oxide.^4^ In addition to protecting against oxidative stress, NRF2 also mitigates nitrosative stress.^38^ Thus, the activation of NRF2 observed in our study may have contributed to the net decline in renal tissue MDA and 3‐NT in ovine sepsis. The unchanged levels of renal tissue iNOS are in accord with those of a previous study, showing that direct renal arterial infusion of the selective iNOS inhibitor, 1400 W, failed to improve renal function in ovine septic AKI.^39^ In contrast, in rodent models of endotoxemia, in which AKI developed over 6 h, there were increases in renal tissue expression of reactive oxygen species.^36^, ^40^ These findings suggest that the sepsis‐induced overproduction in renal tissue oxidative stress reported in experimental sepsis may be species‐, model‐ or time‐dependent. Collectively, our findings indicate that renal oxidative/nitrosative stress is not a prerequisite for the development of septic AKI. Rather, there may be decreased oxidative/nitrosative stress in the setting of Gram‐negative sepsis.

We found that IV administration of tempol mitigated sepsis‐induced hyperemia. The hyperdynamic circulatory state characterized by an increase in cardiac output is a critical driver of the renal hyperemic response in large mammalian models of sepsis, at least over the first 48 h (reviewed in Lankadeva et al.^41^). In a porcine model of sepsis, IVT treatment, at a similar dosage to that used in our current study, significantly reduced sepsis‐induced increases in cardiac output.^42^, ^43^ Therefore, the decreased renal hyperemic response observed in our current study in response to IVT administration is likely to be mediated by attenuation of the hyperdynamic circulatory state. Future studies are required to assess the effects of IV and renal arterial infusions of tempol on cardiac output in this ovine model of Gram‐negative sepsis.

Our study had several strengths. To the best of our knowledge, we provide the first comparison of the effects of a direct renal arterial and IV infusion of tempol on renal macro‐ and micro‐circulatory perfusion, oxygenation, and kidney function. Importantly, our study provides the first evidence that targeted drugs capable of avoiding an early onset of renal medullary hypoxia can prevent the development of AKI in a large mammalian animal model of sepsis with a similar phenotype to human sepsis over a clinically appropriate 24‐h time frame. We controlled for confounding variables such as the volume of parental fluids, timing of sepsis, animal age, and sex. We also performed our experiments in conscious septic sheep, without the confounding effects of anesthetic agents on systemic hemodynamics, sympathetic nerve activity, renal macro‐ and micro‐circulatory perfusion, and PO_2_.^44^, ^45^ All sheep received adequate maintenance fluids (~2000 mL for ~40 kg sheep), which we have previously reported to be sufficient for maintaining central venous pressure and a hyperdynamic circulatory state.^46^ We provide further evidence to substantiate measurement of bladder urinary oxygenation as a reliable estimate of renal medullary hypoxia during septic AKI and in response to therapies that alter medullary tissue oxygenation, which aligns with our recent findings in critically ill humans with and without sepsis.^47^, ^48^, ^49^

We acknowledge several limitations. Gram‐negative sepsis may not fully recapitulate the sepsis‐related pathophysiological changes that occur in response to other infections. Our current experimental design of simultaneously administering tempol from the onset of sepsis does not mimic the clinical scenario, but this was a proof‐of‐concept study designed specifically to determine whether tempol could act within the kidney to prevent sepsis‐induced AKI and to investigate the underlying mechanisms. Although we have no definitive explanation for the disparity between the renal arterial and IV infusions of tempol, our pharmacokinetic study show that the renal arterial route of administration delivers a higher concentration of the nitroxide radical to the kidneys that may reduce renal cortical inflammation and improve renal medullary nitric oxide bioavailability. We were unable to examine the effects of tempol on neurohumoral factors including plasma and urinary nitrate and nitrite concentrations and components of the renin–angiotensin systems, which warrants further investigation. We chose an IV dose of tempol which has been previously reported to mitigate the development of AKI in an anesthetized porcine model of Gram‐negative sepsis.^42^, ^43^ However, future studies are warranted to determine whether higher doses of tempol administered intravenously confer reno‐protective benefits in nonanesthetized sheep subjected to Gram‐negative sepsis. The renal arterial catheter was inserted under direct vision during the abdominal surgery, and it was carefully positioned before the renal arterial bifurcation to enhance homogenous administration of tempol. A limitation is our inability to rule out the potential of streaming during the renal arterial infusion of tempol, which could lead to uneven drug distribution within the kidney.^50^ However, the prevention of renal medullary hypoperfusion and hypoxia achieved with a renal arterial infusion of tempol in the current study indicates that any potential streaming did not counteract drug effectiveness.

In conclusion, using a clinically relevant ovine model of Gram‐negative septic AKI, we found that direct renal arterial infusion of tempol prevented the early onset of renal medullary tissue hypoperfusion and hypoxia and the subsequent development of AKI. Our findings identified potential mechanisms for such an effect, including prevention of overexpression of the inflammatory cytokine TNF‐α in the renal cortex and inhibition of eNOS uncoupling and thus increased bioavailability of nitric oxide in the renal medulla. Further investigation of whether tempol infusion can reverse established septic AKI and whether higher doses of IVT can lead to similar results to those seen with direct renal arterial infusion is warranted.

## 4 MATERIALS AND METHODS

### 4.1 Ethics

All studies fulfilled the Animal Research: Reporting of In Vivo Experiments (ARRIVE) 2.0 criteria.^51^, ^52^ Experimental protocols were approved by the Animal Ethics Committee of the Florey Institute of Neuroscience and Mental Health under the guidelines of the National Health and Medical Research Council of Australia. Prior to experimentation, Merino ewes (40–48 kg body weight) were housed in individual metabolic cages with free access to water and 800 g of oaten chaff daily and were allowed a week of acclimatization to the laboratory environment.

### 4.2 Animal preparation

All sheep underwent two aseptic surgical procedures under isoflurane anesthesia (Isoflo; Zoetis). First, a carotid arterial loop was constructed to facilitate subsequent cannulation for measurement of mean arterial pressure (MAP) and heart rate (HR) and blood sampling.^52^ During the same procedure, the right renal artery, renal vein, and ureter were ligated, and a right unilateral nephrectomy was performed as previously described.^39^ Animals were allowed 4–6 weeks of recovery from the first surgical procedure. The day before the second surgical procedure, the carotid arterial loop and jugular vein were cannulated for IV infusion of tempol and administration of fluids and *E. coli*. In the second surgical procedure, a transit time flow probe (4 mm; Transonic Systems) was placed on the left renal artery to measure RBF, and a Silastic® catheter (ID 0.64 mm OD 1.9 mm; Dow Corning Corporation) was inserted into the left renal artery, as previously described,^39^ to allow direct renal arterial infusion of tempol or vehicle. During the same surgical procedure, the left renal vein was cannulated and custom‐built fiber‐optic probes (Oxford Optronix) were inserted into the renal cortex and medulla, as previously described,^18^, ^52^ for simultaneous measurement of local tissue PO_2_ and laser Doppler flux (as a measure of perfusion). Finally, a Foley catheter (size 12; Euromedical) was inserted into the bladder. Sheep were given intramuscular flunixin meglumine (50 mg; Flunixo; Norbrook) for analgesia and procaine penicillin (900 mg; Ilium, Troy Laboratories) just prior to each surgery and then at 24 and 48 h postoperatively.

### 4.3 Experimental protocol for testing isolated effects of tempol in healthy sheep

In nonanesthetized healthy sheep, baseline measurements commenced 4 days after the second surgical procedure. In five healthy nonseptic sheep, animals received a renal arterial infusion of tempol (3 mg kg^−1^ h^−1^, 4‐Hydroxy‐TEMPO, Sigma‐Aldrich) for 4 h. Animals were allowed 24 h as a washout period and then received an IV infusion of tempol (30 mg kg^−1^ h^−1^) for 4 h. Arterial blood and renal venous blood were collected prior to tempol infusion and then at hourly intervals during the 4‐h continuous infusion of renal arterial and IVT infusion and then at 15 min, 45 min, and 2 h postinfusion to determine pharmacokinetic parameters (see Online Supplement for methodology).

### 4.4 Experimental protocol for testing effects of tempol in septic sheep

An hour preceding the baseline measurement period, a fiber‐optic probe (LAS‐1/O/E, Oxford Optronix) was advanced to the tip of the bladder catheter for continuous measurement of bladder urinary PO_2_ to further validate its utility as a reliable surrogate of renal medullary tissue PO_2_, as previously reported.^8^, ^9^, ^46^, ^47^, ^48^ Urine was collected hourly via a modified fraction collector. Analog signals of MAP, HR, RBF, renal cortical and medullary tissue perfusion, tissue PO_2_, and bladder urinary PO_2_ were continuously recorded at 100 Hz on a computer using a CED Micro 1401 interface with Spike 2 software (Cambridge Electronic Design). RVC was calculated as RBF/MAP.

After 24 h of baseline measurements, Gram‐negative sepsis was induced by IV infusion of an isolate of *E. coli* obtained from a septic patient (Austin Health Pathology). Live *E. coli* was administered as a loading infusion of 2.8 × 10^9^ colony‐forming units (CFU) over 30 min followed by a continuous infusion of 1.26 × 10^9^ CFU h^−1^ for 24 h, as previously described.^7^, ^8^, ^9^ To mitigate hypovolemia, sheep were given a continuous IV infusion of the balanced crystalloid (2 mL kg^−1^ h^−1^), Hartmann's solution (sodium lactate, Baxter Healthcare Pty Ltd), for 24 h from the commencement of live *E. coli* infusion.

From the onset of the infusion of *E. coli*, sheep were randomized to receive either an IV infusion of tempol (30 mg kg^−1^ h^−1^; *n* = 6; 4‐Hydroxy‐TEMPO, Sigma‐Aldrich) or a 10‐fold lower direct renal arterial infusion of tempol (3 mg kg^−1^ h^−1^; *n* = 7) or vehicle (*n* = 7; Hartmann's solution; 6 mL h^−1^) continuously for 24 h. The IV dose of tempol (30 mg kg^−1^ h^−1^) was based on that used in previous studies in an anesthetized porcine model of hyperdynamic Gram‐negative sepsis in which this dose regimen mitigated the development of AKI.^42^, ^43^ The renal arterial dose of tempol (3 mg kg^−1^ h^−1^) used was 10% of the IV dose administered systemically because each kidney receives ~10% of the total cardiac output in sheep.^53^

### 4.5 Periodic measurements in septic sheep

Arterial and renal venous blood samples were collected, at the 24th hour of baseline and then every 4 h during the 24 h infusion of live *E. coli* with and without tempol, for measurement of blood gases and lactate (ABL Systems‐625), TNF‐α and IL‐10 (Kingfisher Biotech Inc.), as previously described.^54^ Arterial blood and urine samples were simultaneously collected for assessment of plasma and urinary MDA (lipid peroxidation assay kit), and creatinine and sodium concentrations (Austin Health Pathology). RDO_2_, RVO_2_, renal oxygen extraction, creatinine clearance, and the fractional excretion of sodium were all calculated using standard formulae, as previously described.^7^, ^8^, ^9^ After 24 h of infusion of *E. coli* (24 h of sepsis), animals were euthanized with pentobarbital (200 mg kg^−1^ IV, Lethobarb; Virbac).

### 4.6 Plasma and renal tissue MDA assays in septic sheep

Malondialdehyde accumulation was assessed with a lipid peroxidation assay kit according to the manufacturer's instructions (Abcam, ab118970). Fluorescence (excitation 532 nm, emission 553 nm) was measured with a CLARIOstar plate reader (BMG Labtech). MDA levels were expressed as nmol mL^−1^ for plasma (100 μL sample) and nmol mg^−1^ of protein for tissue (100 μg of protein from homogenized lysate).

### 4.7 Renal cortical and medullary tissue Western blot analysis in septic sheep

At necropsy, the positions of the fiber‐optic probes within the renal cortex and medulla were confirmed.^18^, ^52^ A 0.5‐cm slice in the transverse plane was then taken from the left kidney, and the cortex and medulla were divided and snap frozen in liquid nitrogen and stored at −80°C for Western blot analysis of 3NT, Nrf2, iNOS, eNOS, phosphorylated eNOS at Ser‐1177 and Thr‐495, TNF‐α, and IL‐10. A separate group of naive, healthy (control) animals (*n* = 5) were euthanized with pentobarbital, and the renal cortical and medullary tissue from their left kidneys were collected for western blot analysis (see online supplement).

### 4.8 Determination of total nitrate and nitrite (NOx) in renal tissue of septic sheep

NOx concentrations were measured in renal cortical and medullary tissue homogenates using a commercially available colorimetric assay kit (Cayman Chemical). Thirty to thirty‐five milligrams of renal cortical or medullary tissue was homogenized in PBS, pH 7.4 (18 mL PBS mg^−1^ of tissue) using a mechanical homogenizer (Polytron PT 2500 E, Kinematica AG) for ~1 min at 15 000 to 17 000 rpm. The homogenate was then centrifuged at 10 000 *g* for 20 min and ultrafiltered using a 10 kDa molecular weight cut‐off (Ultracel®‐10 K, Merck Millipore Ltd) to deproteinize the samples. Nitrate or nitrite standard and tissue filtrate (80 μL) were assayed in duplicates in a 96‐well plate. To determine nitrite, 50 μL of Greiss reagent 1 and Greiss reagent 2 was added and absorbance was read at 540 nm after 10 min incubation. To determine total nitrite, a nitrate reductase and enzyme cofactor were added to the standard and samples and incubated for 3 h before adding the Greiss reagents 1 and 2. The concentrations of NOx were calculated by interpolation from the standard curve. Only the total NOx data are presented because the nitrite determination alone was below the sensitivity limit of the assay.

### 4.9 Statistical analysis

Data are presented as mean ± SD. For variables measured at multiple time points, data were analyzed using a two‐way repeated measures analysis of variance (ANOVA) with factors ‘group’ (*P*_Group_), ‘time’ (*P*_Time_), and their interaction (*P*_Group × Time_). If *P*_Time_ and/or *P*_Group × Time_ was ≤0.05, a Dunnett's post‐test was performed to adjust *p* values for making within‐group multiple comparisons of premorbid baseline Time (0) compared with 4‐, 8‐, 12‐, 16‐, 20‐, and 24‐h time points of sepsis. If *P*_Group_ and/or *p*_Interaction_ was ≤0.05, then a Tukey's post‐test was performed to adjust *p* values for making between‐group multiple comparisons of vehicle, RAT, and IVT groups at each time point. For molecular investigations of renal tissue, between‐group comparisons were made using one‐way ANOVA with a Tukey's post‐test to adjust *p* values for making multiple comparisons between vehicle, RAT, and IVT groups. Lines of best fit were determined by ordinary product regression analysis.^55^ Two‐sided *p* ≤ 0.05 was considered statistically significant. All statistical analyses were performed using either GraphPad (Version 6.0) or Systat (Version 13, Systat).

## AUTHOR CONTRIBUTIONS

Ashenafi H. Betrie, Shuai Ma, Rachel M. Peiris, Connie P. C. Ow, Darius J. R. Lane, and Yugeesh R. Lankadeva were involved in data acquisition, analysis, interpretation, and revision prior to submission. Ashenafi H. Betrie, Scott Ayton, Connie P. C. Ow, Darius J. R. Lane, Adam Southon, Simon R. Bailey, and Yugeesh R. Lankadeva were involved in optimizing and performing molecular assessments on plasma and tissue, interpretation of data, and substantial revision prior to submission. Roger G. Evans and Rinaldo Bellomo were involved in study design, interpretation of data, and substantial revision prior to submission. Yugeesh R. Lankadeva and Clive N. May were involved in conception, hypothesis delineation, study design, analysis, surgical instrumentation, and interpretation of data. Ashenafi H. Betrie, Darius J. R. Lane, and Yugeesh R. Lankadeva designed the pharmacokinetic study, analysis, and interpretation of data. Yugeesh R. Lankadeva wrote the manuscript.

## FUNDING INFORMATION

This study was supported by a grant from the National Health and Medical Research Council of Australia (GNT1188514) and a Young Investigator Medical Research Grant from the Jack Brockhoff Foundation (ID:4178). Y.R.L. was supported by a Future Leader Fellowship from the National Heart Foundation of Australia (FLF105666).

## CONFLICT OF INTEREST STATEMENT

R.G.E. has received consulting fees from Medtronic Inc. All other authors declare no conflicts of interest.

## Supporting information


Figure S1.

Figure S2.

Figure S3.

Figure S4.

Figure S5.

Data S1.

## Data Availability

The data that support the findings of this study are available from the corresponding author upon reasonable request.

## References

[1] Bagshaw SM , George C , Bellomo R , the ADMC . Early acute kidney injury and sepsis: a multicentre evaluation. Crit Care. 2008;12:R47. 18402655 10.1186/cc6863 PMC2447598

[2] Bagshaw SM , Uchino S , Bellomo R , et al. Septic acute kidney injury in critically ill patients: clinical characteristics and outcomes. Clin J Am Soc Nephrol. 2007;2:431‐439. 17699448 10.2215/CJN.03681106

[3] Rhodes A , Evans LE , Alhazzani W , et al. Surviving sepsis campaign: international guidelines for management of sepsis and septic shock: 2016. Intensive Care Med. 2017;43:304‐377. 28101605 10.1007/s00134-017-4683-6

[4] Ow CPC , Trask‐Marino A , Betrie AH , Evans RG , May CN , Lankadeva YR . Targeting oxidative stress in septic acute kidney injury: from theory to practice. J Clin Med. 2021;10:3798. 34501245 10.3390/jcm10173798 PMC8432047

[5] Mittal M , Siddiqui MR , Tran K , Reddy SP , Malik AB . Reactive oxygen species in inflammation and tissue injury. Antioxid Redox Signal. 2014;20:1126‐1167. 23991888 10.1089/ars.2012.5149 PMC3929010

[6] Ma Q . Role of nrf2 in oxidative stress and toxicity. Annu Rev Pharmacol Toxicol. 2013;53:401‐426. 23294312 10.1146/annurev-pharmtox-011112-140320 PMC4680839

[7] Calzavacca P , Evans RG , Bailey M , Bellomo R , May CN . Cortical and medullary tissue perfusion and oxygenation in experimental septic acute kidney injury. Crit Care Med. 2015;43:e431‐e439. 26181218 10.1097/CCM.0000000000001198

[8] Lankadeva YR , Kosaka J , Evans RG , Bellomo R , May CN . Urinary oxygenation as a surrogate marker of medullary oxygenation during angiotensin II therapy in septic acute kidney injury. Crit Care Med. 2018;46:e41‐e48. 29077618 10.1097/CCM.0000000000002797

[9] Lankadeva YR , Kosaka J , Evans RG , Bailey M , Bellomo R , May CN . Intra‐renal and urinary oxygenation during norepinephrine resuscitation in ovine septic acute kidney injury. Kidney Int. 2016;90:100‐108. 27165831 10.1016/j.kint.2016.02.017

[10] Joffre J , Hellman J . Oxidative stress and endothelial dysfunction in sepsis and acute inflammation. Antioxid Redox Signal. 2021;35:1291‐1307. doi:10.1089/ars.2021.0027 33637016

[11] Ow CPC , Ngo JP , Ullah MM , Hilliard LM , Evans RG . Renal hypoxia in kidney disease: cause or consequence? Acta Physiol (Oxf). 2018;222:e12999. 29159875 10.1111/apha.12999

[12] Iannone A , Bini A , Swartz HM , Tomasi A , Vannini V . Metabolism in rat liver microsomes of the nitroxide spin probe tempol. Biochem Pharmacol. 1989;38:2581‐2586. 2764982 10.1016/0006-2952(89)90541-8

[13] Wilcox CS . Effects of tempol and redox‐cycling nitroxides in models of oxidative stress. Pharmacol Ther. 2010;126:119‐145. 20153367 10.1016/j.pharmthera.2010.01.003 PMC2854323

[14] Yin W , Mitra K , Stearns RA , Baillie TA , Kumar S . Conversion of the 2,2,6,6‐tetramethylpiperidine moiety to a 2,2‐dimethylpyrrolidine by cytochrome P450: evidence for a mechanism involving nitroxide radicals and heme iron. Biochemistry. 2004;43:5455‐5466. 15122911 10.1021/bi035944q

[15] Lankadeva YR , Cochrane AD , Marino B , et al. Strategies that improve renal medullary oxygenation during experimental cardiopulmonary bypass may mitigate postoperative acute kidney injury. Kidney Int. 2019;95:1338‐1346. 31005272 10.1016/j.kint.2019.01.032

[16] Lankadeva YR , Evans RG , Cochrane AD , et al. Reversal of renal tissue hypoxia during experimental cardiopulmonary bypass in sheep by increased pump flow and arterial pressure. Acta Physiologica. 2021;231:e13596. 34347356 10.1111/apha.13596

[17] Lankadeva YR , May CN , Cochrane AD , et al. Influence of blood haemoglobin concentration on renal haemodynamics and oxygenation during experimental cardiopulmonary bypass in sheep. Acta Physiol (Oxf). 2021;231:e13583. 33222404 10.1111/apha.13583

[18] Calzavacca P , Evans RG , Bailey M , Lankadeva YR , Bellomo R , May CN . Long‐term measurement of renal cortical and medullary tissue oxygenation and perfusion in unanesthetized sheep. Am J Physiol Regul Integr Comp Physiol. 2015;308:R832‐R839. 25761701 10.1152/ajpregu.00515.2014

[19] Zou AP , Li N , Cowley AW Jr . Production and actions of superoxide in the renal medulla. Hypertension. 2001;37:547‐553. 11230333 10.1161/01.hyp.37.2.547

[20] Wilcox CS , Pearlman A . Chemistry and antihypertensive effects of tempol and other nitroxides. Pharmacol Rev. 2008;60:418‐469. 19112152 10.1124/pr.108.000240 PMC2739999

[21] Rhinehart KL , Pallone TL . Nitric oxide generation by isolated descending vasa recta. Am J Physiol Heart Circ Physiol. 2001;281:H316‐H324. 11406499 10.1152/ajpheart.2001.281.1.H316

[22] Gonçalves GM , Zamboni DS , Câmara NO . The role of innate immunity in septic acute kidney injuries. Shock. 2010;34(Suppl 1):22‐26. 20523275 10.1097/SHK.0b013e3181e7e69e

[23] Xu C , Chang A , Hack BK , Eadon MT , Alper SL , Cunningham PN . TNF‐mediated damage to glomerular endothelium is an important determinant of acute kidney injury in sepsis. Kidney Int. 2014;85:72‐81. 23903370 10.1038/ki.2013.286 PMC3834073

[24] Langenberg C , Gobe G , Hood S , May CN , Bellomo R . Renal histopathology during experimental septic acute kidney injury and recovery*. Crit Care Med. 2014;42:e58‐e67. 24126439 10.1097/CCM.0b013e3182a639da

[25] Silva DAD , Correia TML , Pereira R , da Silva RAA , Augusto O , Queiroz RF . Tempol reduces inflammation and oxidative damage in cigarette smoke‐exposed mice by decreasing neutrophil infiltration and activating the Nrf2 pathway. Chem Biol Interact. 2020;329:109210. 32726580 10.1016/j.cbi.2020.109210

[26] Afjal MA , Abdi SH , Sharma S , et al. Anti‐inflammatory role of tempol (4‐hydroxy‐2,2,6,6‐tetramethylpiperidin‐1‐oxyl) in nephroprotection. Hum Exp Toxicol. 2019;38:713‐723. 30924375 10.1177/0960327119836203

[27] Bendall JK , Alp NJ , Warrick N , et al. Stoichiometric relationships between endothelial tetrahydrobiopterin, endothelial NO synthase (eNOS) activity, and eNOS coupling in vivo: insights from transgenic mice with endothelial‐targeted GTP cyclohydrolase 1 and eNOS overexpression. Circ Res. 2005;97:864‐871. 16179591 10.1161/01.RES.0000187447.03525.72

[28] Fleming I , Fisslthaler B , Dimmeler S , Kemp BE , Busse R . Phosphorylation of Thr(495) regulates Ca(2+)/calmodulin‐dependent endothelial nitric oxide synthase activity. Circ Res. 2001;88:E68‐E75. 11397791 10.1161/hh1101.092677

[29] Lin MI , Fulton D , Babbitt R , et al. Phosphorylation of threonine 497 in endothelial nitric‐oxide synthase coordinates the coupling of L‐arginine metabolism to efficient nitric oxide production. J Biol Chem. 2003;278:44719‐44726. 12952971 10.1074/jbc.M302836200

[30] Aksu U , Ergin B , Bezemer R , et al. Scavenging reactive oxygen species using tempol in the acute phase of renal ischemia/reperfusion and its effects on kidney oxygenation and nitric oxide levels. Intensive Care Med Exp. 2015;3:57. 26215821 10.1186/s40635-015-0057-y PMC4491093

[31] Ergin B , Bezemer R , Kandil A , Demirci‐Tansel C , Ince C . TEMPOL has limited protective effects on renal oxygenation and hemodynamics but reduces kidney damage and inflammation in a rat model of renal ischemia/reperfusion by aortic clamping. J Clin Transl Res. 2015;1:1‐13. PMC6410622 30873445

[32] Iguchi N , Lankadeva Y , Mori T , et al. Furosemide reverses medullary tissue hypoxia in ovine septic acute kidney injury. Am J Physiol Regul Integr Comp Physiol. 2019;317:R232‐R239. 31141418 10.1152/ajpregu.00371.2018

[33] Lorente L , Martín MM , Abreu‐González P , et al. Sustained high serum malondialdehyde levels are associated with severity and mortality in septic patients. Crit Care. 2013;17:R290. 24326199 10.1186/cc13155 PMC4055989

[34] Lorente L , Martín MM , Abreu‐González P , et al. Prognostic value of malondialdehyde serum levels in severe sepsis: a multicenter study. PloS One. 2013;8:e53741. 23341989 10.1371/journal.pone.0053741 PMC3544841

[35] Helan M , Malaska J , Tomandl J , et al. Kinetics of biomarkers of oxidative stress in septic shock: a pilot study. Antioxidants (Basel, Switzerland). 2022;11:640. 35453325 10.3390/antiox11040640 PMC9031382

[36] Feng L‐X , Zhao F , Liu Q , et al. Role of Nrf2 in lipopolysaccharide‐induced acute kidney injury: protection by human umbilical cord blood mononuclear cells. Oxid Med Cell Longev. 2020; 2020:6123459. 32774680 10.1155/2020/6123459 PMC7407026

[37] Wang Y , Feng F , Liu M , Xue J , Huang H . Resveratrol ameliorates sepsis‐induced acute kidney injury in a pediatric rat model via Nrf2 signaling pathway. Exp Ther Med. 2018;16:3233‐3240. 30214546 10.3892/etm.2018.6533 PMC6125985

[38] He X , Ma Q . Disruption of Nrf2 synergizes with high glucose to cause heightened myocardial oxidative stress and severe cardiomyopathy in diabetic mice. J Diabetes Metab. 2012;(Suppl 7);002. 26691239 10.4172/2155-6156.S7-002 PMC4681446

[39] Ishikawa K , Calzavacca P , Bellomo R , Bailey M , May CN . Effect of selective inhibition of renal inducible nitric oxide synthase on renal blood flow and function in experimental hyperdynamic sepsis*. Crit Care Med. 2012;40:2368‐2375. 22622397 10.1097/CCM.0b013e3182514be9

[40] Leach M , Frank S , Olbrich A , Pfeilschifter J , Thiemermann C . Decline in the expression of copper/zinc superoxide dismutase in the kidney of rats with endotoxic shock: effects of the superoxide anion radical scavenger, tempol, on organ injury. Br J Pharmacol. 1998;125:817‐825. 9831920 10.1038/sj.bjp.0702123 PMC1571036

[41] Lankadeva YR , Okazaki N , Evans RG , Bellomo R , May CN . Renal medullary hypoxia: a new therapeutic target for septic acute kidney injury? Semin Nephrol. 2019;39:543‐553. 31836037 10.1016/j.semnephrol.2019.10.004

[42] Matejovic M , Krouzecky A , Martinkova V , et al. Effects of tempol, a free radical scavenger, on long‐term hyperdynamic porcine bacteremia*. Crit Care Med. 2005;33:1057‐1063. 15891336 10.1097/01.ccm.0000162927.94753.63

[43] Matejovic M , Krouzecky A , Rokyta R Jr , et al. Effects of combining inducible nitric oxide synthase inhibitor and radical scavenger during porcine bacteremia. Shock. 2007;27:61‐68. 17172982 10.1097/01.shk.0000235088.53421.6f

[44] Iguchi N , Kosaka J , Booth LC , et al. Renal perfusion, oxygenation, and sympathetic nerve activity during volatile or intravenous general anaesthesia in sheep. Br J Anaesth. 2019;122:342‐349. 30770052 10.1016/j.bja.2018.11.018

[45] Iguchi N , Kosaka J , Iguchi Y , et al. Systemic haemodynamic, renal perfusion and renal oxygenation responses to changes in inspired oxygen fraction during total intravenous or volatile anaesthesia. Br J Anaesth. 2020;125:192‐200. 32563492 10.1016/j.bja.2020.03.033

[46] Lankadeva Y , Kosaka J , Iguchi N , et al. Effects of fluid bolus therapy on renal perfusion, oxygenation, and function in early experimental septic kidney injury. Crit Care Med. 2019;47:e36‐e43. 30394921 10.1097/CCM.0000000000003507

[47] Osawa EA , Cutuli SL , Bitker L , et al. Effect of furosemide on urinary oxygenation in patients with septic shock. Blood Purif. 2019;23:1‐10. 10.1159/000501512 31336370

[48] Plummer MP , Lankadeva YR , Finnis ME , et al. Urinary and renal oxygenation during dexmedetomidine infusion in critically ill adults with mechanistic insights from an ovine model. J Crit Care. 2021;64:74‐81. 33794470 10.1016/j.jcrc.2021.03.004

[49] Osawa EA , Cutuli SL , Yanase F , et al. Effects of changes in inspired oxygen fraction on urinary oxygen tension measurements. Intensive Care Med Exp. 2022;10:52. 36504004 10.1186/s40635-022-00479-y PMC9742069

[50] Parekh N . A novel method for infusing drugs continuously into the renal artery of rats. American Journal of Physiology. 1995;268:F967‐F971. 7771526 10.1152/ajprenal.1995.268.5.F967

[51] Percie du Sert N , Hurst V , Ahluwalia A , et al. The ARRIVE guidelines 2.0: updated guidelines for reporting animal research. PLoS Biol. 2020;18:e3000410. 32663219 10.1371/journal.pbio.3000410 PMC7360023

[52] Lankadeva YR , Kosaka J , Evans RG , May CN . An ovine model for studying the pathophysiology of septic acute kidney injury. Methods Mol Biol. 2018;1717:207‐218. 29468594 10.1007/978-1-4939-7526-6_16

[53] Langenberg C , Wan L , Egi M , May CN , Bellomo R . Renal blood flow in experimental septic acute renal failure. Kidney Int. 2006;69:1996‐2002. 16641923 10.1038/sj.ki.5000440

[54] Lankadeva YR , May CN , McKinley MJ , et al. Sympathetic nerves control bacterial clearance. Sci Rep. 2020;10:15009. 32929135 10.1038/s41598-020-72008-4 PMC7490383

[55] Ludbrook J . A primer for biomedical scientists on how to execute model II linear regression analysis. Clin Exp Pharmacol Physiol. 2012;39:329‐335. 22077731 10.1111/j.1440-1681.2011.05643.x

