# Supplementary figures and images for: Renal arterial infusion of tempol prevents medullary hypoperfusion, hypoxia, and acute kidney injury in ovine Gram‐negative sepsis
Source: Acta Physiol (Oxf). 2023 Aug 7;239(1):e14025. doi: 10.1111/apha.14025 (PMC10909540; doi:10.1111/apha.14025)

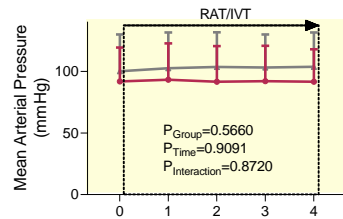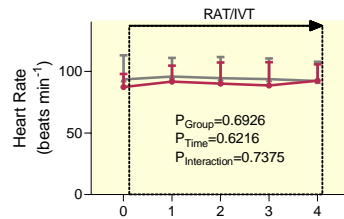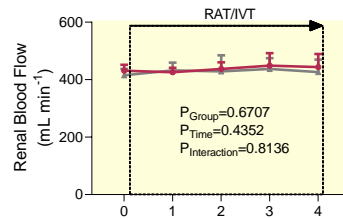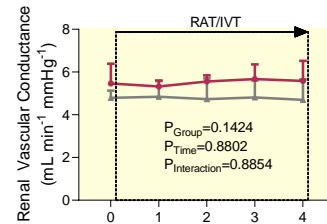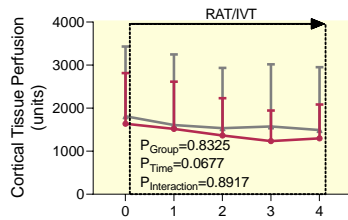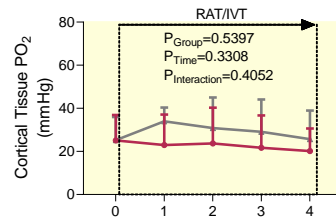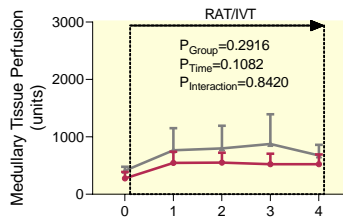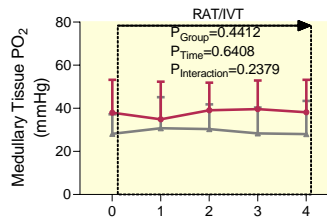

Renal Arterial  
Tempol (RAT)

Intravenous  
Tempol (IVT)

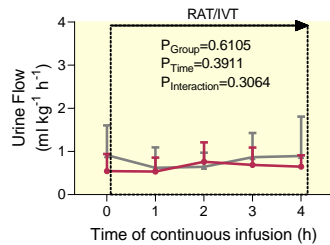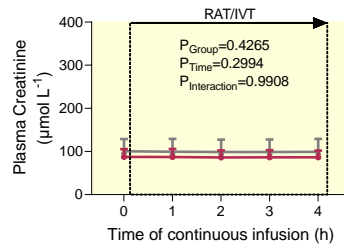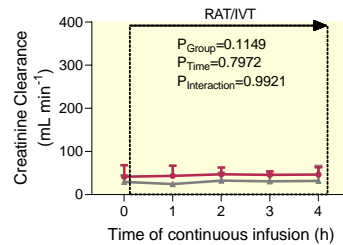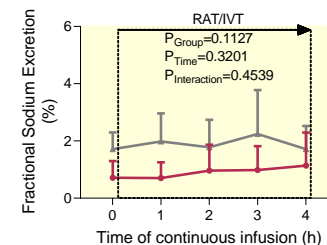

Supplement: Supplementary file 1 — Figure S1. [file APHA-239-e14025-s003.pdf]

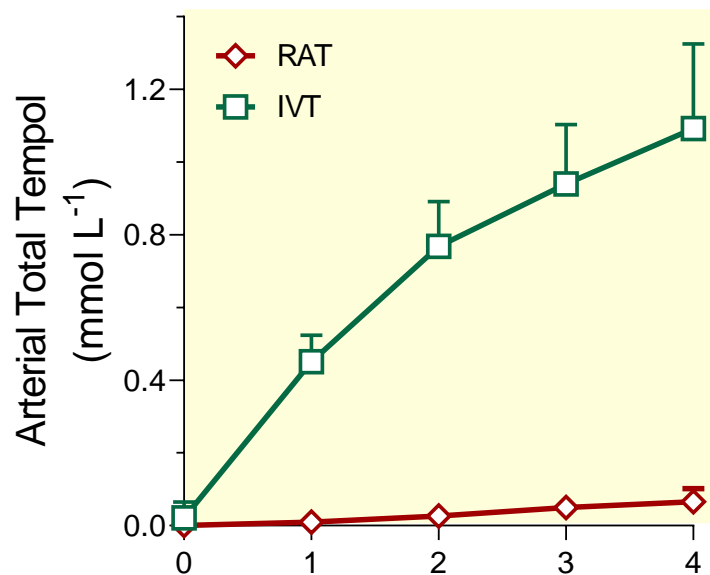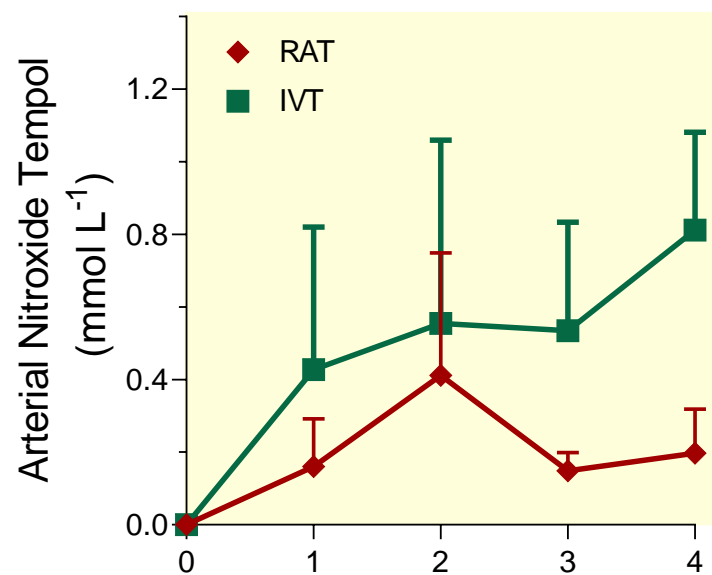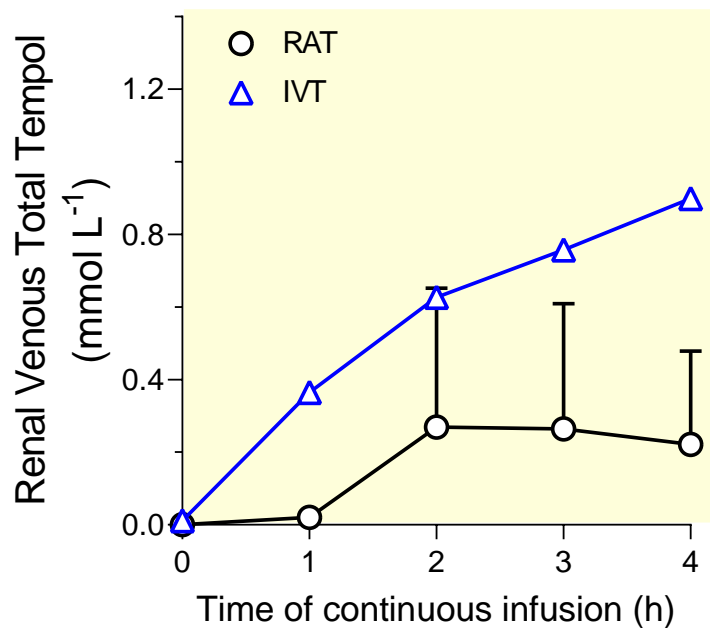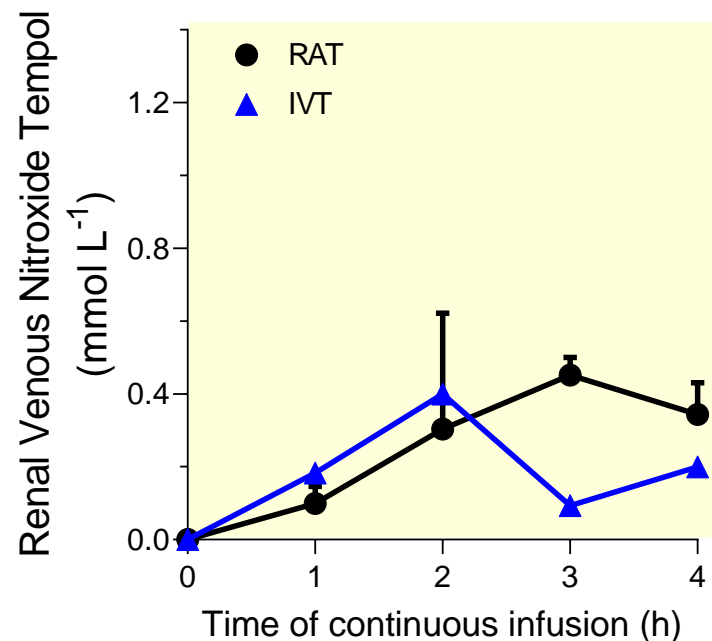

Supplement: Supplementary file 2 — Figure S2. [file APHA-239-e14025-s002.pdf]

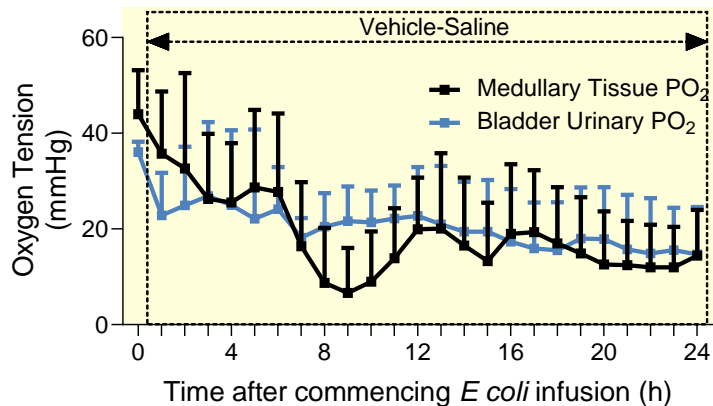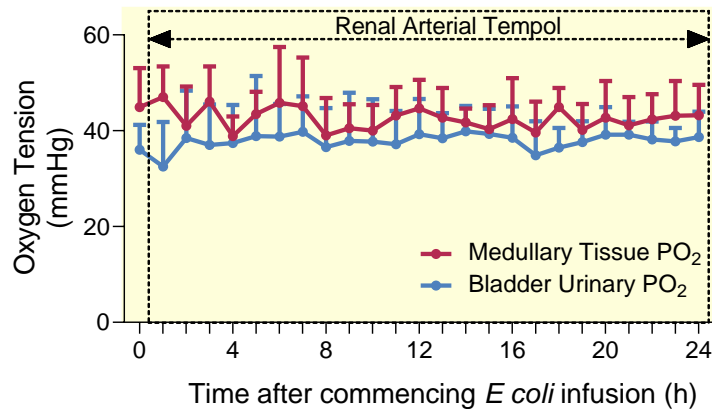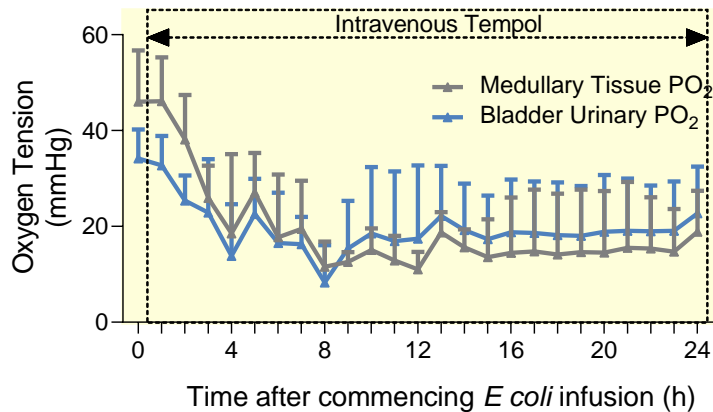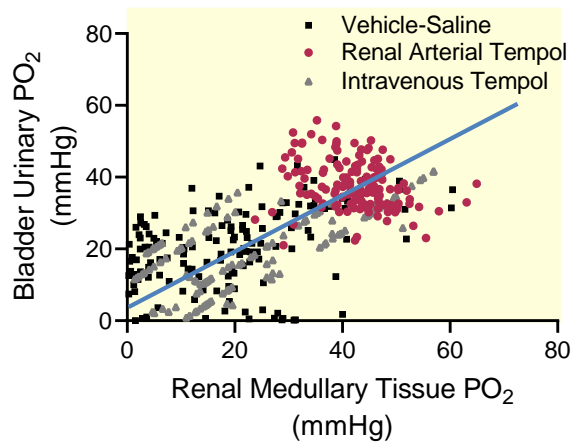

Supplement: Supplementary file 3 — Figure S3. [file APHA-239-e14025-s004.pdf]

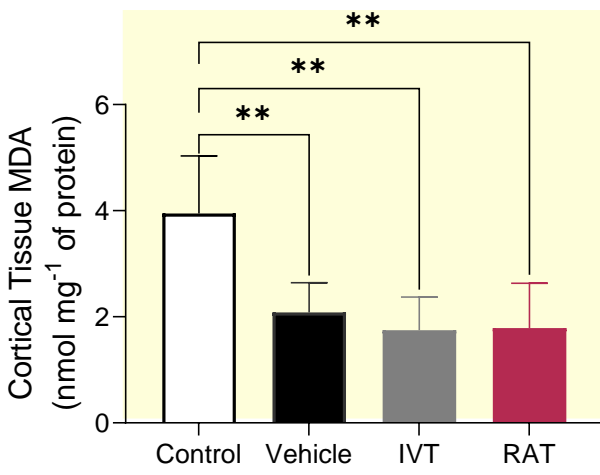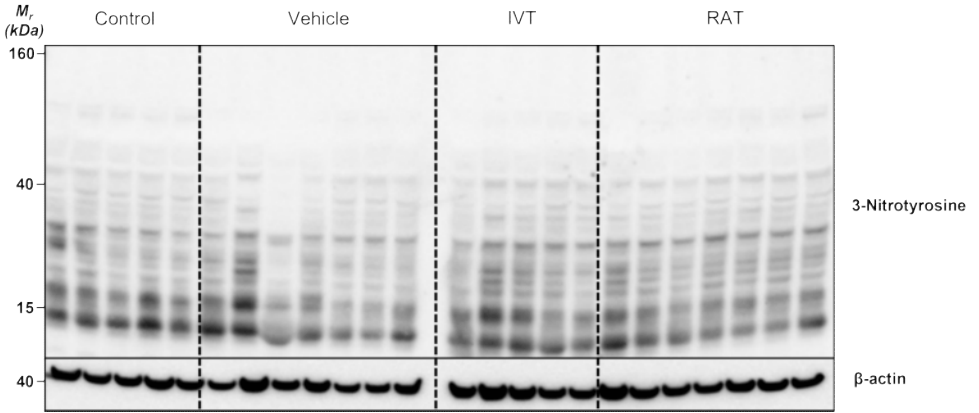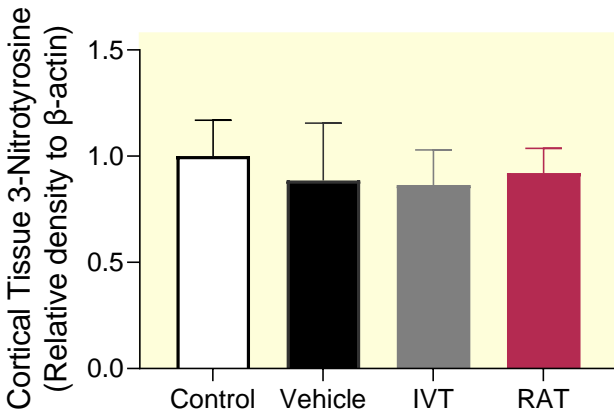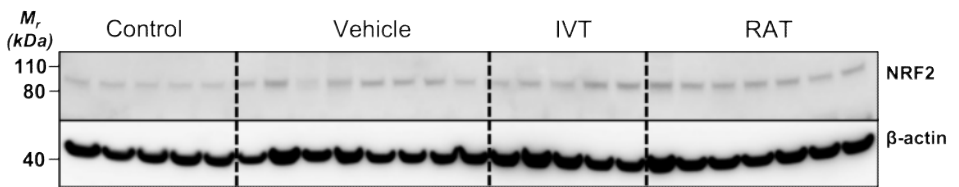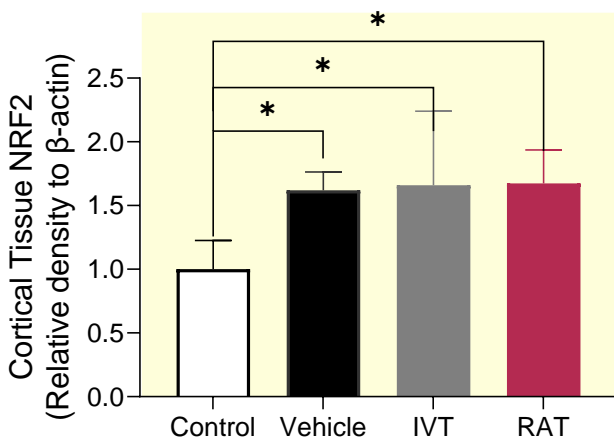

Supplement: Supplementary file 4 — Figure S4. [file APHA-239-e14025-s005.pdf]

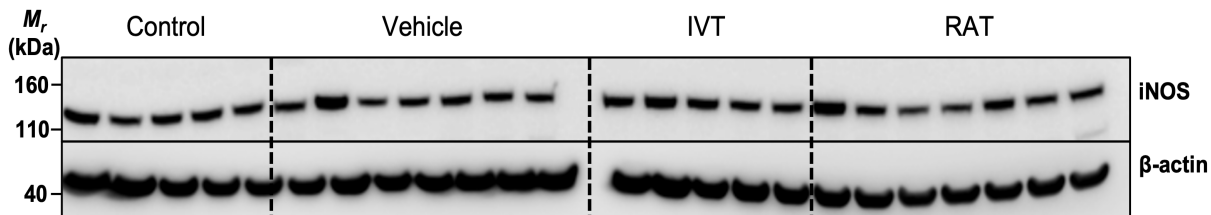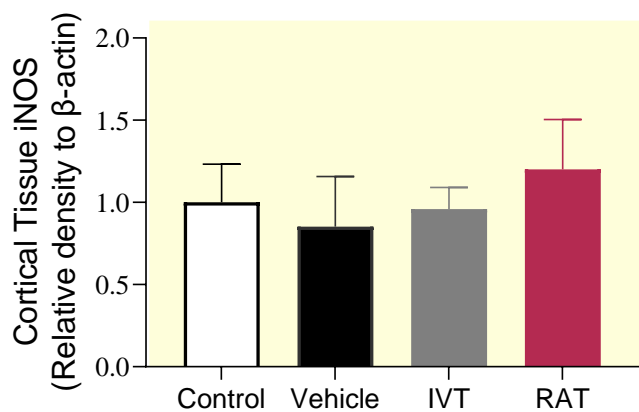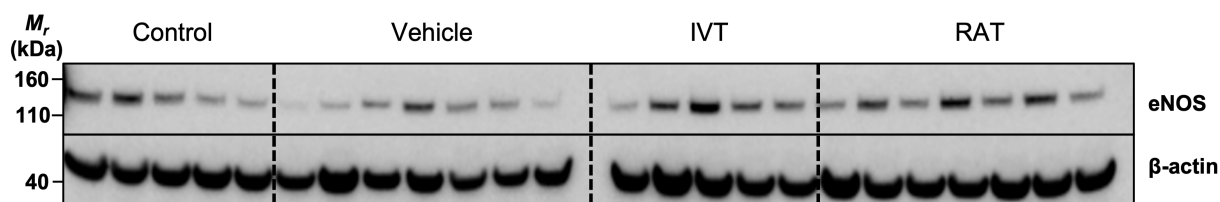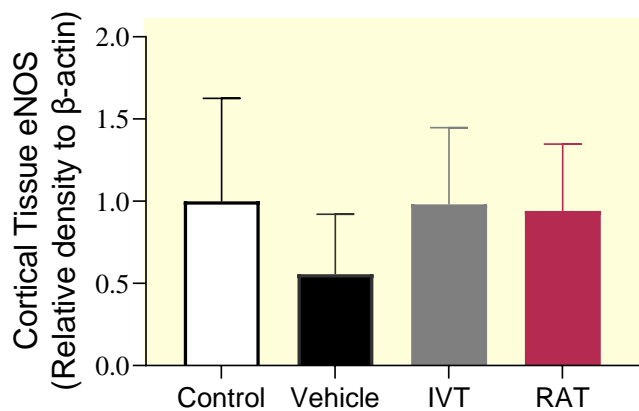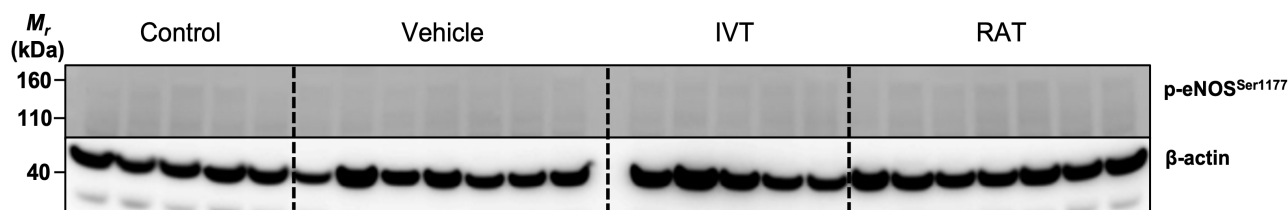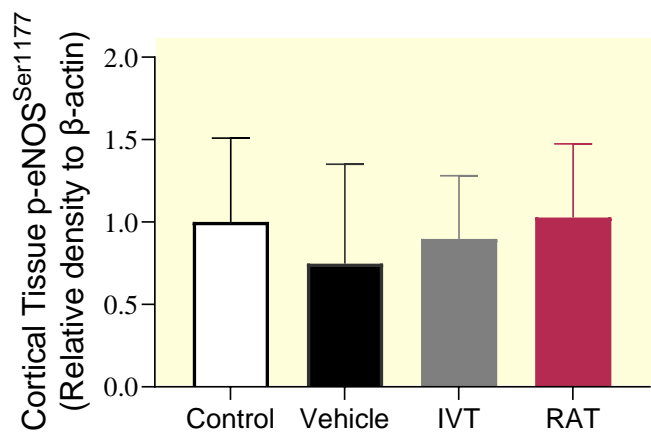

Supplement: Supplementary file 5 — Figure S5. [file APHA-239-e14025-s001.pdf]
