# Supplementary material for: Renal arterial infusion of tempol prevents medullary hypoperfusion, hypoxia, and acute kidney injury in ovine Gram‐negative sepsis
Source: Acta Physiol (Oxf). 2023 Aug 7;239(1):e14025. doi: 10.1111/apha.14025 (PMC10909540; doi:10.1111/apha.14025)
Supplement: Supplementary file 6 — Data S1. [file APHA-239-e14025-s006.docx]

**Supplemental Data**

**Renal arterial infusion of tempol prevents medullary hypoperfusion, hypoxia, and acute kidney injury in ovine Gram-negative sepsis**

Ashenafi H Betrie,^1,2^* Shuai Ma,^1,3^*, Connie PC Ow,^1^ Rachel M Peiris,^1^ Roger G Evans,^1,4^ Scott Ayton,^2^ Darius JR Lane,^2^ Adam Southon,^2^ Simon R Bailey,^5^ Rinaldo Bellomo,^6,7,8,9^ Clive N May,^1,6^* Yugeesh R Lankadeva,^1,6^*

*These authors contributed equally to this work

*^1^Preclinical Critical Care Unit, Florey Institute of Neuroscience and Mental Health, The University of Melbourne, Victoria, Australia; ^2^Translational Neurodegeneration Laboratory, Florey Institute of Neuroscience and Mental Health, The University of Melbourne, Victoria, Australia; ^3^Division of Nephrology, Shanghai Ninth People’s Hospital, Shanghai Jiaotong University School of Medicine, Shanghai, China; ^4^Biomedicine Discovery Institute and Department of Physiology, Monash University, Victoria, Australia; ^5^ Faculty of Veterinary Science, University of Melbourne, VIC, Australia; ^6^Department of Critical Care, Melbourne Medical School, The University of Melbourne, Victoria, Australia; ^7^Australian and New Zealand Intensive Care Research Centre, Monash University, Melbourne, Australia; ^8^Deaprtment of Intensive Care, Austin Hospital, Melbourne, Australia, ^9^Department of Intensive Care, Royal Melbourne Hospital, Melbourne, Australia*

**Supplemental Methods:**

**Western blot analysis**

Renal cortical and medullary tissue (~100 mg) were homogenized in ice-cold radioimmunoprecipitation assay buffer (RIPA: 50 mM tris, 150 mM NaCl, 1% Triton X-100, 0.5% sodium deoxycholate and 0.1% sodium dodecyl sulfate, pH 7.4) containing EDTA-free protease inhibitor cocktail (1:50, Roche, Mannheim, Germany) and phosphatase inhibitor cocktail (1:1000, Roche). Samples were then sonicated for 5-8 strokes at 70% amplitude with 0.3 s pulse ON and pulse OFF, keeping the samples at ~4ºC, and centrifuged for 30 min (13200 rpm) at 4° C. Total protein concentration in the supernatant was determined by the bicinchoninic acid protein assay (Pierce, Rockford, IL, USA). Equal protein concentrations (40 µg) were loaded to 4-12% bis-Tris gels with NuPAGE MES running buffer (Invitrogen, Carlsbad, CA, USA).

After completion of the electrophoresis, the gels were transferred to PVDF membranes using an iBlot dry blotting system (Invitrogen). Membranes were blocked with 5% BSA in 1% tris-buffered saline with Tween (TBST; 10 mM Tris HCL, 150 mM NaCl and 1% Tween 20). Blots were then incubated with primary antibodies overnight (phosphorylated-eNOS (serine (Ser) 1177), Cell Signaling Technology #9571, 1:500; phosphorylated-eNOS (threonine (Thr)-495), Cell Signaling Technology #9574, 1:500; eNOS/NOS type III, BD Transduction Laboratories, #610297, 1:1000; iNOS, Abcam, #ab204017, 1: 1000; 3-nitrotyrosine, Abcam, #ab61392, 1:5000, NRF2 D1Z9C, Cell Signaling Technology, #127215, 1:1000, TNF-α (Kingfisher Biotech Inc, #KP1454V, 1:1000 and IL-10, Kingfisher, # KP1255B, 1:1000 and beta actin, Sigma, #A5441, 1:5000). Blots were then washed with TBST three times (10 min on a rocker) followed by a secondary HRP-conjugated antibody (1:2000 – 1:5000). An enhanced chemiluminescence detection system (GE Healthcare, Uppsala, Sweden) was used for developing the gel which was visualized using Fujifilm LAS-3000. Protein bands were quantified and normalized against the housekeeping protein β-actin using Image J 1.48.

**Determination of total Tempol by reaction with 2,2-diphenyl-1-picrylhydrazyl (DPPH)**

An assay was developed and employed to quantify total 4-hydroxy-tempo (Tempol, ranging from 0.01 to 1 mM) in plasma samples obtained from healthy sheep. This assay is characterized by high sensitivity and selectivity for tempol, leveraging the specific reaction between 2,2-diphenyl-1-picrylhydrazyl (DPPH) and the 4-hydroxy group of tempol in a methanol solution. Initially, 500 µL aliquots of sheep plasma were prepared by diluting with an equal volume of 250 mM Tris-HCl buffer (pH 8.4). The samples were then ultrafiltered through a 10 kDa molecular-weight cut-off membrane (Ultracel®-10K, Merck Millipore Ltd, Carrigtwohill co Cork, Ireland) by centrifugation at 15,000 × g for 20 minutes at 4°C. Subsequently, 20 µL of each ultrafiltrate were transferred into 2 mL Eppendorf tubes, followed by the addition of 1.2 mL of 0.22 mM DPPH dissolved in methanol. Standards, encompassing a known range of tempol concentrations, were subjected to the identical treatment. The tubes were vortexed briefly to ensure mixing and then incubated at room temperature (approximately 25°C) in the dark for 24 hours. Post-incubation, the absorbance of DPPH was measured at 517 nm and 410 nm using either a spectrophotometer with a cuvette holder or a microplate reader equipped for 96-well plates (1 cm pathlength specified). The ratio of the absorbance at 410 nm to that at 517 nm was calculated for each sample, which exhibited a positive linear dose-response up to a ratio of approximately 2.0. Tempol concentrations in the diluted and ultrafiltered plasma samples were determined by interpolating from a standard curve generated with known concentrations of tempol. The measurements were first corrected for the 0 mM Tempol blank, and subsequently adjusted for background values corresponding to baseline samples that contained no tempol (typically <1% of the peak plasma tempol reading). Data analysis and curve fitting were conducted using GraphPad Prism software, version 9.0.

**Determination of Tempol nitroxide radical by UV spectrophotometry**

The concentration of tempol was determined using ultraviolet (UV) spectrophotometry. Absorbance spectra for varying standard concentrations of tempol, ranging from 0.1 mM to 2 mM, as well as for the plasma samples, were recorded in duplicate. Measurements were performed using a 96-well quartz microplate (manufactured by Hellma GmbH & Co. KG, Mullheim, Germany) compatible with UV absorbance readings. The absorbance spectra were acquired, focusing on the characteristic peak of tempol in its nitroxide radical form, which has a maximum absorbance (λ_max_) at 242 nm. This peak absorbance at 242 nm in the standards was utilized to estimate the concentration of tempol in the samples. Prior to measurement, plasma samples were first diluted and ultrafiltered using a 10 kDa molecular weight cut-off membrane (Merck) to deproteinize the samples and reduce interference. Tempol concentrations in these processed plasma samples were then calculated by interpolating from a standard curve of peak absorbance for known standard concentrations of tempol (GraphPad Prism software, version 9.0). The absorbance values of the baseline samples, which were obtained from each animal before tempol infusion, served as the blank for normalizing the measurements. This ensured that any inherent absorbance in the samples not attributed to Tempol was accounted for in the analysis.

**Tempol pharmacokinetics**

In 5 healthy sheep, tempol (30 mg kg^-1^ h^-1^) was infused intravenously for 4 hours. Plasma was collected from systemic blood at baseline and every hour. At 4 hours, the infusion was stopped, and further plasma was collected at 15 min, 45 min and 2 hours. Samples were diluted using TBS (1:1 v/v) to preserve tempol. Tempol values were determined by UV spectrophotometer or DPPH assay (see above) to estimate the nitroxide radical and the metabolites (hydroxylamine and oxoammonium). The averaged data from 4 animals (1 animal excluded due to this data being an outlier to curve of best fit) was used to analyse pharmacokinetic parameters using a one-compartmental model using the PKSolver 2.0 add-in in MS Excel [1].

**Supplemental results:**

**Relationship between bladder urinary and medullary tissue oxygenation**

The temporal changes in bladder urinary PO_2_ closely reflected those of renal medullary tissue PO_2_ (Supplementary Figure 3). Bladder urinary PO_2_ fell during the 24 h period of infusion of *E. coli* in the sheep treated with either vehicle (36 ± 2 to 15 ± 10 mmHg; P=0.006) or intravenous tempol (36 ± 3 to 21 ± 8 mmHg; P=0.02) but not in sheep receiving a renal arterial infusion of tempol (36 ± 5 to 39 ± 5 mmHg; P>0.1). Regression analysis demonstrated a moderate linear correlation between bladder urinary and renal medullary tissue PO_2_ (r^2^ = 0.456).

**Supplemental Figure 1:** Systemic and renal hemodynamics, intra-renal perfusion and oxygenation and kidney functional indices during intravenous and renal arterial tempol in healthy sheep.

Time courses of mean arterial pressure, heart rate, renal blood flow, renal vascular conductance, renal cortical and medullary tissue perfusion and oxygenation, urine flow, plasma creatinine, creatinine clearance and fractional sodium excretion during 4 hours of continuous infusion of intravenous (30 mg kg^-1^ h^-1^) or renal arterial (3 mg kg^-1^ h^-1^) tempol in healthy non-septic sheep. Values are presented as mean ± standard deviation. P values were determined from a two-way repeated measures ANOVA with factors Group (P_Group_), time (P_Time_) and their interaction (P_Interaction_).

**Supplemental Figure 2:** Concentrations of tempol nitroxide radical and total tempol determined from UV absorbance and DPPH assays.

Total tempol (nitroxide, hydroxylamine and oxoammonium forms) or the nitroxide radical concentrations were measured from plasma taken from arterial (n=5) or renal venous blood (n=3). Measurements were done before and after infusing the nitroxide radical, tempol, via renal arterial or carotid arterial catheters for 4 hours. Values are presented as mean ± standard deviation.

**Supplemental Figure 3:** Relationship between medullary and bladder urinary oxygen tension during renal arterial infusion of tempol or vehicle or intravenous infusion of tempol during 24 hours of ovine sepsis.

Time-courses of renal medullary tissue (n=6; open symbols) and bladder urinary (n=6; closed symbols) oxygen tension (PO_2_) during renal arterial infusion of vehicle, intravenous infusion of tempol, and renal arterial infusion of tempol. Each point is between-animal mean ± SD of a 60 min average. Scatterplot of the relationship between medullary tissue and urinary PO_2_ during renal infusion of tempol or vehicle or systemic infusion of tempol. Symbols show 60 min averages for 6 sheep, with different symbols for the three treatments. The line of best fit, in the format of Y = a +b*X was determined using ordinary least-products regression. The regression coefficients and 95% confidence limits are a = 3.548 (1.616-5.480), b = 0.786 (0.728-0.845), with a Pearson Product-Moment Correlation Coefficient (r^2^) of 0.456.

**Supplemental Figure 4:** Protein expression of renal cortical tissue oxidative stress, nitrosative stress and markers of antioxidant defense mechanisms.

Renal cortical malonaldehyde, renal cortical 3-nitrotyrosine and renal cortical nuclear factor-erythroid related factor 2 protein expression in tissue derived from naïve sheep (control; n=5) and from septic sheep treated either with a renal arterial infusion of tempol (RAT; n=7) or vehicle (n=7) or an intravenous infusion of tempol (IVT; n=5) during the 24 h period of sepsis. Values are presented as mean ± standard deviation of protein expression relative to the expression of β-actin. Data were analyzed using a one-way ANOVA and *P<0.05, **P<0.01 and ***P<0.001 are derived from Tukey’s post-test for comparisons between the different treatment groups.

**Supplemental Figure 5:** Renal cortical protein expression of isoforms of nitric oxide synthase isoforms.

Inducible nitric oxide synthase, endothelial nitric oxide synthase and eNOS phosphorylated at serine at position 1177 (p-eNOS^Ser-1177^) in renal cortical tissue derived from naïve sheep (control; n=5) and from septic sheep treated either with a renal arterial infusion of tempol (RAT; n=7) or vehicle (n=7) or an intravenous infusion of tempol (IVT; n=5) during the 24 h period of sepsis. Values are presented as mean ± standard deviation of protein expression relative to the expression of β-actin. Data were analyzed using a one-way ANOVA and *P<0.05, **P<0.01 and ***P<0.001 are derived from Tukey’s post-test for comparisons between the different treatment groups.

**Supplemental Table 1: One-compartmental model pharmacokinetic analysis of total tempol and nitroxide before, during and after intravenous tempol infusion in healthy sheep.**

| **Averaged raw data for one-compartment PK modelling** | | | | | **Pharmacokinetic parameters from one-compartment PK analysis** | | | | | |
| --- | --- | --- | --- | --- | --- | --- | --- | --- | --- | --- |
| **Tempol radical (nitroxide)- measured by peak UV absorbance at 242 nm wavelength** | | | | | **PK parameters for Tempol radical (nitroxide)** | | | **PK parameters of total Tempol (nitroxide + other forms)** | | |
| **Time (h)** | **Conc (µmol L^-1^)** | **%CV** | ***ln*(Conc)** | **n** | **Parameter** | **Unit** | **Value** | **Parameter** | **Value** |  |
| 0 | 0 | 0 | 0 | 4 | A | μmol L^-1^ | 2677.2594 | A | 2010.6199 |  |
| 1 | 573.969 | 65.6877 | 6.3526 | 4 | k_10_ | h^-1^ | 0.7270 | k_10_ | 0.4011 |  |
| 2 | 714.6155 | 71.4279 | 6.5717 | 4 | t_1/2_ | h | 0.9534 | t1/2 | 1.7283 |  |
| 3 | 565.3795 | 57.7680 | 6.3375 | 4 | V | (mg kg^-1^)/(μmol L^-1^) | 0.0448 | V | 0.0597 |  |
| 4 | 1089.651 | 46.5421 | 6.9936 | 4 | CL | (mg kg^-1^)/( μmol L^-1^) h^-1^ | 0.0326 | CL | 0.0239 |  |
| 4.25 | 691.321 | 25.0298 | 6.5386 | 4 | Tmax | h | 4 | Tmax | 4 |  |
| 4.75 | 443.5795 | 28.9967 | 6.0949 | 4 | Cmax | μmol L^-1^ | 870.3509 | Cmax | 1001.3457 |  |
| 6 | 286.1875 | 88.4475 | 5.6566 | 4 | Css | μmol L^-1^ | 920.5914 | Css | 1253.3142 |  |
|  |  |  |  |  | AUC0-∞ | μmol L^-1^*h | 3682.3655 | AUC0-∞ | 5013.2566 |  |
| **Total Tempol (nitroxide + other forms) measured by DPPH assay** | | | |  | AUMC | μmol L^-1^*h^2^ | 5064.8119 | AUMC | 12499.9963 |  |
| **Time** | **Conc (µmol L^-1^)** | **%CV** | ***ln*(Conc)** |  | MRT | h | 1.3754 | MRT | 2.4934 |  |
| 0 | 0.85 | 144.2476 | -0.1625 | 4 | **Diagnostics** | | | | | |
| 1 | 441.1 | 18.2330 | 6.0893 | 4 | r_obs-pre_ | 0.9052 | | r_obs-pre_ | 0.9983 |  |
| 2 | 719.05 | 15.7188 | 6.5779 | 4 |  |  | |  |  |  |
| 3 | 865.975 | 15.7242 | 6.7639 | 4 | SS | 132740.61 | | SS | 3375.28 |  |
| 4 | 985.225 | 16.3122 | 6.8929 | 4 | R^2^ | 0.9572 | | R^2^ | 0.9992 |  |
| 4.25 | 877.15 | 18.1401 | 6.7767 | 4 | SE | 148.7395 | | SE | 23.7181 |  |
| 4.75 | 758.375 | 20.7600 | 6.6312 | 4 | AIC | 98.3692 | | AIC | 68.9939 |  |
| 6 | 468.475 | 25.7455 | 6.1495 | 4 | SC | 98.5281 | | SC | 69.1528 |  |

k_10_, elimination rate constant; t_1/2_, half-life; V, volume of distribution; CL, clearance; MRT: mean residence time, Css, steady state concentration; AUMC, area under the first moment curve; SS, sum of squares of residuals; SE, standard error of weighted residuals; AIC, Akaike’s information criterion; SC, Schwarz criterion.

**Supplemental Table 2: Statistical analysis from repeated measures two-way ANOVA.**

Results from a two-way repeated measures ANOVA with factors ‘group’ (P_Group_), ‘time’ (P_Time_) and their interaction (P_GroupxTime_). If P_Time_ and/or P_GroupxTime_ was ≤0.05, a Dunnett’s post-test was performed to adjust P values for making within-group multiple comparisons of pre-morbid baseline Time (0) compared with the 4-, 8-, 12-, 16-, 20- and 24-hour time-points of sepsis. If P_Group_ and/or P_Interaction_ was ≤0.05, then a Tukey’s post-test was performed to adjust P values for comparisons between vehicle, renal arterial tempol and intravenous tempol at each of the sepsis-time points.

| **Figure 1: Mean Arterial Pressure (mmHg)** | |
| --- | --- |
| Two-way ANOVA Results | P_Group_ = 0.0624  **P_Time_ < 0.0001**  P_GroupxTime_ = 0.1164 |
| Renal arterial tempol (RAT) infusion | Adjusted P values from Dunnett’s test |
| 0 vs. 4 hr  0 vs. 8 hr  0 vs. 12 hr  0 vs. 16 hr  0 vs. 20 hr  0 vs. 24 hr | 0.0362*  0.0124*  0.5308  0.4653  0.2123  0.0262* |
| Intravenous tempol (IVT) infusion | Adjusted P values from Dunnett’s test |
| 0 vs. 4 hr  0 vs. 8 hr  0 vs. 12 hr  0 vs. 16 hr  0 vs. 20 hr  0 vs. 24 hr | 0.0030**  >0.9999  0.1080  0.1248  0.0364*  0.0100* |
| Vehicle (Veh) Infusion | Adjusted P values from Dunnett’s test |
| 0 vs. 4 hr  0 vs. 8 hr  0 vs. 12 hr  0 vs. 16 hr  0 vs. 20 hr  0 vs. 24 hr | 0.0086**  0.7458  0.5031  0.0630  0.0122*  0.0024** |

| **Figure 1: Heart Rate (beats min^-1^)** | |
| --- | --- |
| Two-way ANOVA Results | P_Group_ = 0.3138  **P_Time_ < 0.0001**  P_GroupxTime_ = 0.2906 |
| Renal arterial tempol (RAT) infusion | Adjusted P values from Dunnett’s test |
| 0 vs. 4 hr  0 vs. 8 hr  0 vs. 12 hr  0 vs. 16 hr  0 vs. 20 hr  0 vs. 24 hr | 0.4317  0.0060**  0.0006***  0.0023**  0.0006***  0.0003** |
| Intravenous tempol (IVT) infusion | Adjusted P values from Dunnett’s test |
| 0 vs. 4 hr  0 vs. 8 hr  0 vs. 12 hr  0 vs. 16 hr  0 vs. 20 hr  0 vs. 24 hr | 0.1245  0.0008***  0.0101*  0.0188*  0.0137*  0.0014** |
| Vehicle (Veh) Infusion | Adjusted P values from Dunnett’s test |
| 0 vs. 4 hr  0 vs. 8 hr  0 vs. 12 hr  0 vs. 16 hr  0 vs. 20 hr  0 vs. 24 hr | 0.7687  0.0001***  0.0003***  <0.0001****  <0.0001****  <0.0001**** |

| **Figure 1: Renal Blood Flow (mL min^-1^)** | | | |
| --- | --- | --- | --- |
| Two-way ANOVA Results | **P_Group_ = 0.0126**  **P_Time_ < 0.0001**  P_GroupxTime_ = 0.0849 | | |
| **Time analysis** | | **Group analysis** | |
| Renal arterial tempol (RAT) infusion | Adjusted P values from Dunnett’s test | Group Multiple comparison | Adjusted P values from Tukey’s test |
| 0 vs. 4 hr  0 vs. 8 hr  0 vs. 12 hr  0 vs. 16 hr  0 vs. 20 hr  0 vs. 24 hr | 0.0316*  0.0127*  0.0062**  0.0403*  0.0312*  0.0124* | 0 hr (RAT vs IVT)  0 hr (RAT vs Veh)  0 hr (IVT vs Veh) | 0.1049  0.9412  0.4344 |
|  |  | 4 hr (RAT vs IVT)  4 hr (RAT vs Veh)  4 hr (IVT vs Veh) | 0.0114*  0.2236  0.0915 |
| Intravenous tempol (IVT) infusion | Adjusted P Values from Dunnett’s test | 8 hr (RAT vs IVT)  8 hr (RAT vs Veh)  8 hr (IVT vs Veh) | 0.0222*  0.2803  0.1532 |
| 0 vs. 4 hr  0 vs. 8 hr  0 vs. 12 hr  0 vs. 16 hr  0 vs. 20 hr  0 vs. 24 hr | 0.1423  0.0002***  0.0202*  0.0886  0.0205*  0.0088** | 12 hr (RAT vs IVT)  12 hr (RAT vs Veh)  12 hr (IVT vs Veh) | 0.0079**  0.2952  0.1699 |
|  |  | 16 hr (RAT vs IVT)  16 hr (RAT vs Veh)  16 hr (IVT vs Veh) | 0.0880  0.3575  0.6185 |
| Vehicle (Veh) Infusion | Adjusted P Values from Dunnett’s test | 20 hr (RAT vs IVT)  20 hr (RAT vs Veh)  20 hr (IVT vs Veh) | 0.0461*  0.3496  0.3491 |
| 0 vs. 4 hr  0 vs. 8 hr  0 vs. 12 hr  0 vs. 16 hr  0 vs. 20 hr  0 vs. 24 hr | 0.0298*  0.0019**  0.0080**  0.0184*  0.0096**  0.0306* | 24 hr (RAT vs IVT)  24 hr (RAT vs Veh)  24 hr (IVT vs Veh) | 0.0193*  0.3300  0.4818 |

| **Figure 1: Renal Vascular Conductance (mL min^-1^mmHg^-1^)** | |
| --- | --- |
| Two-way ANOVA Results | P_Group_ = 0.3494  **P_Time_ < 0.0001**  P_GroupxTime_ = 0.4294 |
| Renal arterial tempol (RAT) infusion | Adjusted P values from Dunnett’s test |
| 0 vs. 4 hr  0 vs. 8 hr  0 vs. 12 hr  0 vs. 16 hr  0 vs. 20 hr  0 vs. 24 hr | 0.8897  0.3754  0.0272*  0.0611  0.0766  0.0360* |
| Intravenous tempol (IVT) infusion | Adjusted P values from Dunnett’s test |
| 0 vs. 4 hr  0 vs. 8 hr  0 vs. 12 hr  0 vs. 16 hr  0 vs. 20 hr  0 vs. 24 hr | 0.6491  0.0005***  0.0440*  0.0496*  0.0001***  0.0003*** |
| Vehicle (Veh) Infusion | Adjusted P values from Dunnett’s test |
| 0 vs. 4 hr  0 vs. 8 hr  0 vs. 12 hr  0 vs. 16 hr  0 vs. 20 hr  0 vs. 24 hr | 0.9911  0.0733  0.0180*  0.0084**  0.0048**  0.0026** |

| **Figure 2: Renal Oxygen Delivery (mL O_2_ min^-1^)** | | | |
| --- | --- | --- | --- |
| Two-way ANOVA Results | **P_Group_ = 0.0126**  **P_Time_ < 0.0001**  P_GroupxTime_ = 0.0849 | | |
| **Time analysis** | | **Group analysis** | |
| Renal arterial tempol (RAT) infusion | Adjusted P values from Dunnett’s test | Group Multiple comparison | Adjusted P values from Tukey’s test |
| 0 vs. 4 hr  0 vs. 8 hr  0 vs. 12 hr  0 vs. 16 hr  0 vs. 20 hr  0 vs. 24 hr | 0.0848  0.0428*  0.0203*  0.0808  0.0597  0.0297* | 0 hr (RAT vs IVT)  0 hr (RAT vs Veh)  0 hr (IVT vs Veh) | 0.0988  0.7629  0.0051** |
|  |  | 4 hr (RAT vs IVT)  4 hr (RAT vs Veh)  4 hr (IVT vs Veh) | 0.0611  0.9861  0.0002*** |
| Intravenous tempol (IVT) infusion | Adjusted P Values from Dunnett’s test | 8 hr (RAT vs IVT)  8 hr (RAT vs Veh)  8 hr (IVT vs Veh) | 0.0711  0.9996  0.0003*** |
| 0 vs. 4 hr  0 vs. 8 hr  0 vs. 12 hr  0 vs. 16 hr  0 vs. 20 hr  0 vs. 24 hr | 0.2305  0.0366*  0.0223*  0.8199  0.3594  0.1625 | 12 hr (RAT vs IVT)  12 hr (RAT vs Veh)  12 hr (IVT vs Veh) | 0.0847  0.8020  0.0494* |
|  |  | 16 hr (RAT vs IVT)  16 hr (RAT vs Veh)  16 hr (IVT vs Veh) | 0.0994  0.9734  0.0010*** |
| Vehicle (Veh) Infusion | Adjusted P Values from Dunnett’s test | 20 hr (RAT vs IVT)  20 hr (RAT vs Veh)  20 hr (IVT vs Veh) | 0.1144  0.9963  0.0062** |
| 0 vs. 4 hr  0 vs. 8 hr  0 vs. 12 hr  0 vs. 16 hr  0 vs. 20 hr  0 vs. 24 hr | 0.0050**  0.0003***  0.0255*  0.0001***  0.0062**  0.0035** | 24 hr (RAT vs IVT)  24 hr (RAT vs Veh)  24 hr (IVT vs Veh) | 0.1092  0.8711  0.0080** |

| **Figure 2: Renal Oxygen Consumption (mL O_2_ min^-1^)** | |
| --- | --- |
| Two-way ANOVA Results | P_Group_ = 0.8097  **P_Time_ = 0.0244**  P_GroupxTime_ = 0.6437 |
| Renal arterial tempol (RAT) infusion | Adjusted P values from Dunnett’s test |
| 0 vs. 4 hr  0 vs. 8 hr  0 vs. 12 hr  0 vs. 16 hr  0 vs. 20 hr  0 vs. 24 hr | 0.9998  0.9957  0.9941  0.9306  0.9927  0.3130 |
| Intravenous tempol (IVT) infusion | Adjusted P values from Dunnett’s test |
| 0 vs. 4 hr  0 vs. 8 hr  0 vs. 12 hr  0 vs. 16 hr  0 vs. 20 hr  0 vs. 24 hr | 0.2397  0.4772  0.6700  0.1075  0.1755  0.0144* |
| Vehicle (Veh) Infusion | Adjusted P values from Dunnett’s test |
| 0 vs. 4 hr  0 vs. 8 hr  0 vs. 12 hr  0 vs. 16 hr  0 vs. 20 hr  0 vs. 24 hr | 0.0877  0.7210  0.4640  0.8300  0.3560  0.4129 |

| **Figure 2: Renal Oxygen Extraction (%)** | |
| --- | --- |
| Two-way ANOVA Results | P_Group_ = 0.1224  **P_Time_ = 0.0006**  P_GroupxTime_ = 0.5786 |
| Renal arterial tempol (RAT) infusion | Adjusted P values from Dunnett’s test |
| 0 vs. 4 hr  0 vs. 8 hr  0 vs. 12 hr  0 vs. 16 hr  0 vs. 20 hr  0 vs. 24 hr | 0.1617  0.1475  0.4872  0.6238  0.6988  0.1807 |
| Intravenous tempol (IVT) infusion | Adjusted P values from Dunnett’s test |
| 0 vs. 4 hr  0 vs. 8 hr  0 vs. 12 hr  0 vs. 16 hr  0 vs. 20 hr  0 vs. 24 hr | 0.1070  0.0192*  0.9925  0.2939  0.5976  0.1560 |
| Vehicle (Veh) Infusion | Adjusted P values from Dunnett’s test |
| 0 vs. 4 hr  0 vs. 8 hr  0 vs. 12 hr  0 vs. 16 hr  0 vs. 20 hr  0 vs. 24 hr | 0.0392*  0.3205  0.0736  0.8041  0.1663  0.2715 |

| **Figure 3: Medullary Tissue Perfusion (Units)** | | | |
| --- | --- | --- | --- |
| Two-way ANOVA Results | **P_Group_ < 0.0001**  **P_Time_ < 0.0001**  **P_GroupxTime_ < 0.0001** | | |
| **Time analysis** | | **Group analysis** | |
| Renal arterial tempol (RAT) infusion | Adjusted P values from Dunnett’s test | Group Multiple comparison | Adjusted P values from Tukey’s test |
| 0 vs. 4 hr  0 vs. 8 hr  0 vs. 12 hr  0 vs. 16 hr  0 vs. 20 hr  0 vs. 24 hr | 0.3682  0.9757  0.9999  0.9023  0.5166  0.5497 | 0 hr (RAT vs IVT)  0 hr (RAT vs Veh)  0 hr (IVT vs Veh) | 0.9511  0.7849  0.8212 |
|  |  | 4 hr (RAT vs IVT)  4 hr (RAT vs Veh)  4 hr (IVT vs Veh) | 0.0849  0.0175*  0.8059 |
| Intravenous tempol (IVT) infusion | Adjusted P Values from Dunnett’s test | 8 hr (RAT vs IVT)  8 hr (RAT vs Veh)  8 hr (IVT vs Veh) | 0.0735  0.0067**  0.6315 |
| 0 vs. 4 hr  0 vs. 8 hr  0 vs. 12 hr  0 vs. 16 hr  0 vs. 20 hr  0 vs. 24 hr | 0.0291*  0.0255*  0.0067**  0.0002***  0.0002***  <0.0001**** | 12 hr (RAT vs IVT)  12 hr (RAT vs Veh)  12 hr (IVT vs Veh) | 0.0010***  <0.0001****  0.9980 |
|  |  | 16 hr (RAT vs IVT)  16 hr (RAT vs Veh)  16 hr (IVT vs Veh) | 0.0074**  0.0091**  0.9321 |
| Vehicle (Veh) Infusion | Adjusted P Values from Dunnett’s test | 20 hr (RAT vs IVT)  20 hr (RAT vs Veh)  20 hr (IVT vs Veh) | 0.0086**  0.0111*  0.8912 |
| 0 vs. 4 hr  0 vs. 8 hr  0 vs. 12 hr  0 vs. 16 hr  0 vs. 20 hr  0 vs. 24 hr | 0.0171*  0.0028**  0.0023**  0.0015**  0.0011**  0.0026** | 24 hr (RAT vs IVT)  24 hr (RAT vs Veh)  24 hr (IVT vs Veh) | 0.0003***  0.0002***  0.9940 |

| **Figure 3: Medullary Tissue PO_2_ (mmHg)** | | | |
| --- | --- | --- | --- |
| Two-way ANOVA Results | **P_Group_< 0.0001**  **P_Time_ < 0.0001**  **P_GroupxTime_ < 0.0001** | | |
| **Time analysis** | | **Group analysis** | |
| Renal arterial tempol (RAT) infusion | Adjusted P values from Dunnett’s test | Group Multiple comparison | Adjusted P values from Tukey’s test |
| 0 vs. 4 hr  0 vs. 8 hr  0 vs. 12 hr  0 vs. 16 hr  0 vs. 20 hr  0 vs. 24 hr | 0.1862  0.6079  0.9996  0.5560  0.3804  0.4906 | 0 hr (RAT vs IVT)  0 hr (RAT vs Veh)  0 hr (IVT vs Veh) | 0.9376  0.9831  0.8673 |
|  |  | 4 hr (RAT vs IVT)  4 hr (RAT vs Veh)  4 hr (IVT vs Veh) | <0.0001****  0.0153*  0.0427* |
| Intravenous tempol (IVT) infusion | Adjusted P Values from Dunnett’s test | 8 hr (RAT vs IVT)  8 hr (RAT vs Veh)  8 hr (IVT vs Veh) | <0.0001****  0.0004***  0.4830 |
| 0 vs. 4 hr  0 vs. 8 hr  0 vs. 12 hr  0 vs. 16 hr  0 vs. 20 hr  0 vs. 24 hr | 0.0008***  <0.0001****  <0.0001****  0.0158*  0.0210*  0.0137* | 12 hr (RAT vs IVT)  12 hr (RAT vs Veh)  12 hr (IVT vs Veh) | <0.0001****  0.0005***  0.1234 |
|  |  | 16 hr (RAT vs IVT)  16 hr (RAT vs Veh)  16 hr (IVT vs Veh) | 0.0007***  0.0036**  0.8416 |
| Vehicle (Veh) Infusion | Adjusted P Values from Dunnett’s test | 20 hr (RAT vs IVT)  20 hr (RAT vs Veh)  20 hr (IVT vs Veh) | 0.0020**  <0.0001****  0.9667 |
| 0 vs. 4 hr  0 vs. 8 hr  0 vs. 12 hr  0 vs. 16 hr  0 vs. 20 hr  0 vs. 24 hr | 0.0037**  0.0006***  0.0087**  0.0210*  0.0013**  0.0006*** | 24 hr (RAT vs IVT)  24 hr (RAT vs Veh)  24 hr (IVT vs Veh) | 0.0003***  <0.0001****  0.3449 |

| **Figure 3: Cortical Tissue Perfusion (Units)** | |
| --- | --- |
| Two-way ANOVA Results | P_Group_ = 0.8449  **P_Time_ < 0.0001**  P_GroupxTime_= 0.5745 |
| Renal arterial tempol (RAT) infusion | Adjusted P values from Dunnett’s test |
| 0 vs. 4 hr  0 vs. 8 hr  0 vs. 12 hr  0 vs. 16 hr  0 vs. 20 hr  0 vs. 24 hr | 0.9983  0.9997  0.1972  0.5923  0.2352  0.0261* |
| Intravenous tempol (IVT) infusion | Adjusted P values from Dunnett’s test |
| 0 vs. 4 hr  0 vs. 8 hr  0 vs. 12 hr  0 vs. 16 hr  0 vs. 20 hr  0 vs. 24 hr | 0.9757  0.0531  0.1126  0.2675  0.2695  0.0456* |
| Vehicle (Veh) Infusion | Adjusted P values from Dunnett’s test |
| 0 vs. 4 hr  0 vs. 8 hr  0 vs. 12 hr  0 vs. 16 hr  0 vs. 20 hr  0 vs. 24 hr | 0.2715  0.1599  0.1829  0.1179  0.2294  0.0438* |

| **Figure 3: Cortical Tissue PO_2_ (mmHg)** | |
| --- | --- |
| Two-way ANOVA Results | P_Group_ = 0.3611  **P_Time_ = 0.0183**  P_GroupxTime_ = 0.5570 |
| Renal arterial tempol (RAT) infusion | Adjusted P values from Dunnett’s test |
| 0 vs. 4 hr  0 vs. 8 hr  0 vs. 12 hr  0 vs. 16 hr  0 vs. 20 hr  0 vs. 24 hr | 0.9911  0.7094  0.9998  0.9863  0.9255  0.6260 |
| Intravenous tempol (IVT) infusion | Adjusted P values from Dunnett’s test |
| 0 vs. 4 hr  0 vs. 8 hr  0 vs. 12 hr  0 vs. 16 hr  0 vs. 20 hr  0 vs. 24 hr | 0.7727  0.4961  0.4580  0.3046  0.3889  0.2707 |
| Vehicle (Veh) Infusion | Adjusted P values from Dunnett’s test |
| 0 vs. 4 hr  0 vs. 8 hr  0 vs. 12 hr  0 vs. 16 hr  0 vs. 20 hr  0 vs. 24 hr | 0.9660  0.9999  0.8298  0.5272  0.7470  0.9420 |

| **Figure 4: Plasma Creatinine (μmol L^-1^)** | | | |
| --- | --- | --- | --- |
| Two-way ANOVA Results | **P_Group_ = 0.0038**  **P_Time_ <0.0001**  **P_GroupxTime_ <0.0001** | | |
| **Time analysis** | | **Group analysis** | |
| Renal arterial tempol (RAT) infusion | Adjusted P values from Dunnett’s test | Group Multiple comparison | Adjusted P values from Tukey’s test |
| 0 vs. 4 hr  0 vs. 8 hr  0 vs. 12 hr  0 vs. 16 hr  0 vs. 20 hr  0 vs. 24 hr | 0.0316*  0.1491  0.0070**  0.0132*  0.0157*  0.1240 | 0 hr (RAT vs IVT)  0 hr (RAT vs Veh)  0 hr (IVT vs Veh) | 0.9420  0.9820  0.7992 |
|  |  | 4 hr (RAT vs IVT)  4 hr (RAT vs Veh)  4 hr (IVT vs Veh) | 0.7706  0.5615  0.9765 |
| Intravenous tempol (IVT) infusion | Adjusted P Values from Dunnett’s test | 8 hr (RAT vs IVT)  8 hr (RAT vs Veh)  8 hr (IVT vs Veh) | 0.1465  0.0286*  0.3379 |
| 0 vs. 4 hr  0 vs. 8 hr  0 vs. 12 hr  0 vs. 16 hr  0 vs. 20 hr  0 vs. 24 hr | 0.1547  0.0009***  0.0001***  0.0003***  0.0010**  0.0001*** | 12 hr (RAT vs IVT)  12 hr (RAT vs Veh)  12 hr (IVT vs Veh) | 0.0436*  0.0363*  0.2300 |
|  |  | 16 hr (RAT vs IVT)  16 hr (RAT vs Veh)  16 hr (IVT vs Veh) | 0.0058**  0.0293*  0.2934 |
| Vehicle (Veh) Infusion | Adjusted P Values from Dunnett’s test | 20 hr (RAT vs IVT)  20 hr (RAT vs Veh)  20 hr (IVT vs Veh) | 0.0107*  0.0194*  0.2705 |
| 0 vs. 4 hr  0 vs. 8 hr  0 vs. 12 hr  0 vs. 16 hr  0 vs. 20 hr  0 vs. 24 hr | 0.2992  0.0303*  0.0306*  0.0279*  0.0225*  0.0319* | 24 hr (RAT vs IVT)  24 hr (RAT vs Veh)  24 hr (IVT vs Veh) | 0.0014**  0.0220*  0.3208 |

| **Figure 4: Creatinine Clearance (mL min^-1^)** | | | |
| --- | --- | --- | --- |
| Two-way ANOVA Results | **P_Group_ = 0.0012**  **P_Time_ <0.0001**  **P_GroupxTime_ <0.0001** | | |
| **Time analysis** | | **Group analysis** | |
| Renal arterial tempol (RAT) infusion | Adjusted P values from Dunnett’s test | Group Multiple comparison | Adjusted P values from Tukey’s test |
| 0 vs. 4 hr  0 vs. 8 hr  0 vs. 12 hr  0 vs. 16 hr  0 vs. 20 hr  0 vs. 24 hr | 0.5288  0.9841  0.3705  0.4980  0.6413  0.9613 | 0 hr (RAT vs IVT)  0 hr (RAT vs Veh)  0 hr (IVT vs Veh) | 0.1017  0.8768  0.5985 |
|  |  | 4 hr (RAT vs IVT)  4 hr (RAT vs Veh)  4 hr (IVT vs Veh) | 0.9931  0.5517  0.7577 |
| Intravenous tempol (IVT) infusion | Adjusted P Values from Dunnett’s test | 8 hr (RAT vs IVT)  8 hr (RAT vs Veh)  8 hr (IVT vs Veh) | 0.8002  0.7621  0.9331 |
| 0 vs. 4 hr  0 vs. 8 hr  0 vs. 12 hr  0 vs. 16 hr  0 vs. 20 hr  0 vs. 24 hr | 0.9984  0.3577  <0.0001****  0.0008***  0.0019**  0.0071** | 12 hr (RAT vs IVT)  12 hr (RAT vs Veh)  12 hr (IVT vs Veh) | 0.1851  0.0945  0.6699 |
|  |  | 16 hr (RAT vs IVT)  16 hr (RAT vs Veh)  16 hr (IVT vs Veh) | 0.0212*  0.0021**  0.0811 |
| Vehicle (Veh) Infusion | Adjusted P Values from Dunnett’s test | 20 hr (RAT vs IVT)  20 hr (RAT vs Veh)  20 hr (IVT vs Veh) | 0.0041**  <0.0001****  0.0137* |
| 0 vs. 4 hr  0 vs. 8 hr  0 vs. 12 hr  0 vs. 16 hr  0 vs. 20 hr  0 vs. 24 hr | 0.9313  0.8962  0.0074**  0.0005***  0.0004***  0.0003*** | 24 hr (RAT vs IVT)  24 hr (RAT vs Veh)  24 hr (IVT vs Veh) | 0.0156*  0.0005***  0.0106* |

| **Figure 4: Urine flow (mL kg^-1^ h^-1^)** | | | |
| --- | --- | --- | --- |
| Two-way ANOVA Results | **P_Group_ = 0.0144**  **P_Time_ <0.0001**  **P_GroupxTime_ = 0.2665** | | |
| **Time analysis** | | **Group analysis** | |
| Renal arterial tempol (RAT) infusion | Adjusted P values from Dunnett’s test | Group Multiple comparison | Adjusted P values from Tukey’s test |
| 0 vs. 4 hr  0 vs. 8 hr  0 vs. 12 hr  0 vs. 16 hr  0 vs. 20 hr  0 vs. 24 hr | 0.0107*  >0.9999  >0.9999  0.9980  0.6568  0.5166 | 0 hr (RAT vs IVT)  0 hr (RAT vs Veh)  0 hr (IVT vs Veh) | 0.9632  0.5019  0.5901 |
|  |  | 4 hr (RAT vs IVT)  4 hr (RAT vs Veh)  4 hr (IVT vs Veh) | 0.6028  0.5722  0.9713 |
| Intravenous tempol (IVT) infusion | Adjusted P Values from Dunnett’s test | 8 hr (RAT vs IVT)  8 hr (RAT vs Veh)  8 hr (IVT vs Veh) | 0.1217  0.6183  0.6852 |
| 0 vs. 4 hr  0 vs. 8 hr  0 vs. 12 hr  0 vs. 16 hr  0 vs. 20 hr  0 vs. 24 hr | 0.3213  0.1611  0.1296  0.1281  0.1229  0.1614 | 12 hr (RAT vs IVT)  12 hr (RAT vs Veh)  12 hr (IVT vs Veh) | <0.0001****  0.4582  0.4244 |
|  |  | 16 hr (RAT vs IVT)  16 hr (RAT vs Veh)  16 hr (IVT vs Veh) | 0.0174*  0.0206*  0.9859 |
| Vehicle (Veh) Infusion | Adjusted P Values from Dunnett’s test | 20 hr (RAT vs IVT)  20 hr (RAT vs Veh)  20 hr (IVT vs Veh) | 0.0083**  0.0057**  0.7653 |
| 0 vs. 4 hr  0 vs. 8 hr  0 vs. 12 hr  0 vs. 16 hr  0 vs. 20 hr  0 vs. 24 hr | 0.4619  0.9997  0.9604  0.0016**  0.0035**  0.0015** | 24 hr (RAT vs IVT)  24 hr (RAT vs Veh)  24 hr (IVT vs Veh) | 0.0029**  0.0012**  0.1401 |

| **Figure 4: Fractional Sodium Excretion (%)** | | | |
| --- | --- | --- | --- |
| Two-way ANOVA Results | **P_Group_ = 0.0144**  **P_Time_ <0.0001**  **P_GroupxTime_ = 0.0551** | | |
| **Time analysis** | | **Group analysis** | |
| Renal arterial tempol (RAT) infusion | Adjusted P values from Dunnett’s test | Group Multiple comparison | Adjusted P values from Tukey’s test |
| 0 vs. 4 hr  0 vs. 8 hr  0 vs. 12 hr  0 vs. 16 hr  0 vs. 20 hr  0 vs. 24 hr | 0.0219*  0.7413  0.3374  0.4718  0.8148  0.9546 | 0 hr (RAT vs IVT)  0 hr (RAT vs Veh)  0 hr (IVT vs Veh) | 0.7197  0.7500  0.9981 |
|  |  | 4 hr (RAT vs IVT)  4 hr (RAT vs Veh)  4 hr (IVT vs Veh) | 0.1677  0.1268  0.9332 |
| Intravenous tempol (IVT) infusion | Adjusted P Values from Dunnett’s test | 8 hr (RAT vs IVT)  8 hr (RAT vs Veh)  8 hr (IVT vs Veh) | 0.6591  0.5803  0.9983 |
| 0 vs. 4 hr  0 vs. 8 hr  0 vs. 12 hr  0 vs. 16 hr  0 vs. 20 hr  0 vs. 24 hr | 0.4866  0.1799  0.0070**  0.0201*  0.0018**  0.0202* | 12 hr (RAT vs IVT)  12 hr (RAT vs Veh)  12 hr (IVT vs Veh) | 0.4031  0.9132  0.8236 |
|  |  | 16 hr (RAT vs IVT)  16 hr (RAT vs Veh)  16 hr (IVT vs Veh) | 0.5906  0.5057  0.9721 |
| Vehicle (Veh) Infusion | Adjusted P Values from Dunnett’s test | 20 hr (RAT vs IVT)  20 hr (RAT vs Veh)  20 hr (IVT vs Veh) | 0.0407*  0.0627  0.9781 |
| 0 vs. 4 hr  0 vs. 8 hr  0 vs. 12 hr  0 vs. 16 hr  0 vs. 20 hr  0 vs. 24 hr | 0.4076  0.3616  0.3648  0.4978  0.1006  0.0492* | 24 hr (RAT vs IVT)  24 hr (RAT vs Veh)  24 hr (IVT vs Veh) | 0.0112*  0.0018**  0.3028 |

| **Figure 5: Plasma MDA (nmol/mL)** | |
| --- | --- |
| Two-way ANOVA Results  (Mixed-effects analysis) | P_Group_ = 0.7697  **P_Time_ < 0.0001**  P_GroupxTime_ = 0.9923 |
| Renal arterial tempol (RAT) infusion | Adjusted P values from Dunnett’s test |
| 0 vs. 4 hr  0 vs. 8 hr  0 vs. 12 hr  0 vs. 16 hr  0 vs. 20 hr  0 vs. 24 hr | 0.9982  0.1005  0.1331  0.2372  0.2108  0.0738 |
| Intravenous tempol (IVT) infusion | Adjusted P values from Dunnett’s test |
| 0 vs. 4 hr  0 vs. 8 hr  0 vs. 12 hr  0 vs. 16 hr  0 vs. 20 hr  0 vs. 24 hr | 0.5423  0.1108  0.1828  0.0693  0.1644  0.0742 |
| Vehicle (Veh) Infusion | Adjusted P values from Dunnett’s test |
| 0 vs. 4 hr  0 vs. 8 hr  0 vs. 12 hr  0 vs. 16 hr  0 vs. 20 hr  0 vs. 24 hr | 0.8626  0.2582  0.2724  0.2824  0.6693  0.3293 |

| **Figure 6: Plasma Tumour Necrosis Factor-α (ng/ml)** | | | |
| --- | --- | --- | --- |
| Two-way ANOVA Results | **P_Group_ = 0.0069**  **P_Time_ < 0.0001**  **P_GroupxTime_ < 0.0001** | | |
| **Time analysis** | | **Group analysis** | |
| Renal arterial tempol (RAT) infusion | Adjusted P values from Dunnett’s test | Group Multiple comparison | Adjusted P values from Tukey’s test |
| 0 vs. 4 hr  0 vs. 8 hr  0 vs. 12 hr  0 vs. 16 hr  0 vs. 20 hr  0 vs. 24 hr | 0.0652  0.2030  0.4268  0.2645  0.5400  0.9985 | 0 hr (RAT vs IVT)  0 hr (RAT vs Veh)  0 hr (IVT vs Veh) | 0.8543  0.9761  0.9789 |
|  |  | 4 hr (RAT vs IVT)  4 hr (RAT vs Veh)  4 hr (IVT vs Veh) | 0.9680  0.0186*  0.0147* |
| Intravenous tempol (IVT) infusion | Adjusted P Values from Dunnett’s test | 8 hr (RAT vs IVT)  8 hr (RAT vs Veh)  8 hr (IVT vs Veh) | 0.8236  0.0114*  0.0074** |
| 0 vs. 4 hr  0 vs. 8 hr  0 vs. 12 hr  0 vs. 16 hr  0 vs. 20 hr  0 vs. 24 hr | 0.3448  0.2573  0.0744  0.5499  0.8344  0.9960 | 12 hr (RAT vs IVT)  12 hr (RAT vs Veh)  12 hr (IVT vs Veh) | 0.5312  0.2255  0.1096 |
|  |  | 16 hr (RAT vs IVT)  16 hr (RAT vs Veh)  16 hr (IVT vs Veh) | 0.8523  0.6347  0.4455 |
| Vehicle (Veh) Infusion | Adjusted P Values from Dunnett’s test | 20 hr (RAT vs IVT)  20 hr (RAT vs Veh)  20 hr (IVT vs Veh) | 0.6078  0.9381  0.5779 |
| 0 vs. 4 hr  0 vs. 8 hr  0 vs. 12 hr  0 vs. 16 hr  0 vs. 20 hr  0 vs. 24 hr | 0.0092**  0.0049**  0.0853  0.1168  0.0872  >0.9999 | 24 hr (RAT vs IVT)  24 hr (RAT vs Veh)  24 hr (IVT vs Veh) | 0.9834  0.9811  0.8953 |

| **Figure 6: Plasma Interleukin-10 (ng/mL)** | |
| --- | --- |
| Two-way ANOVA Results | P_Group_ = 0.9315  **P_Time_ < 0.0001**  P_GroupxTime_ = 0.1585 |
| Renal arterial tempol (RAT) infusion | Adjusted P values from Dunnett’s test |
| 0 vs. 4 hr  0 vs. 8 hr  0 vs. 12 hr  0 vs. 16 hr  0 vs. 20 hr  0 vs. 24 hr | 0.0212*  0.0167*  0.1960  0.2011  0.0130*  0.0041** |
| Intravenous tempol (IVT) infusion | Adjusted P values from Dunnett’s test |
| 0 vs. 4 hr  0 vs. 8 hr  0 vs. 12 hr  0 vs. 16 hr  0 vs. 20 hr  0 vs. 24 hr | 0.0505  0.0273*  0.0394*  0.0531  0.0208*  0.0105* |
| Vehicle (Veh) Infusion | Adjusted P values from Dunnett’s test |
| 0 vs. 4 hr  0 vs. 8 hr  0 vs. 12 hr  0 vs. 16 hr  0 vs. 20 hr  0 vs. 24 hr | 0.0303*  0.0327*  0.0141*  0.0041**  0.0238*  0.0253* |
